# Melandryoside attenuates steatohepatitis and fibrosis by restoring PPM1A-mediated SMAD signaling termination

**DOI:** 10.3389/fphar.2026.1880176

**Published:** 2026-08-05

**Authors:** Yo Han Lee, Youngsang Nam, Kieun Park, Haneul Ji, Ji Won Kim, Sooyeon Cho, Na Gyeong Kim, Kwangho Kim, Jiwon Yoo, Ji Young Lee, Chang-Hwan Bae, Eunsoo Kim, Eun Seo Jang, Won Kon Kim, Hwayoung Yun, Seoung Rak Lee, Jun Hong Park, Jang Hyun Choi

**Affiliations:** 1 Department of Biological Sciences, Ulsan National Institute of Science and Technology (UNIST), Ulsan, Republic of Korea; 2 College of Pharmacy and Research Institute for Drug Development, Pusan National University, Busan, Republic of Korea; 3 Herbal Medicine Resources Research Center, Korea Institute of Oriental Medicine, Naju, Jeollanam-do, Republic of Korea; 4 Metabolic Regulation Research Center, Korea Research Institute of Bioscience and Biotechnology (KRIBB), Daejeon, Republic of Korea; 5 College of Veterinary Medicine, Jeonbuk National University, Iksan-si, Jeollanam-do, Republic of Korea; 6 Metadium Therapeutics, Ulsan, Republic of Korea

**Keywords:** *de novo* lipogenesis, fibrosis, inflammation, MASH, Melandrii Herba, PPM1A, ROS production, Smad2

## Abstract

**Background:**

Metabolic dysfunction-associated steatohepatitis (MASH) is characterized by hepatic steatosis, inflammation, and fibrosis, yet effective pharmacological therapies remain limited. Melandrii Herba, a traditional East Asian medicinal herb used to improve blood circulation and relieve inflammatory disorders, has reported anti-inflammatory activity. However, its therapeutic potential and active constituents in MASH remain unclear.

**Purpose:**

This study investigated the hepatoprotective and anti-fibrotic effects of Melandrii Herba ethanol extract (MHE) and its purified constituent melandryoside in MASH and explored the underlying mechanisms.

**Methods:**

Palmitic acid-challenged HepG2 hepatocytes and TGF-β-stimulated HSC-LX2 cells were used to evaluate metabolic and fibrogenic stress responses. *In vivo* efficacy was examined in choline-deficient, L-amino acid-defined, high-fat diet (CDAHFD)-fed mice, with MHE administered therapeutically after disease establishment (weeks 8–12) under continued dietary challenge. Interactome analysis, co-immunoprecipitation, and PPM1A loss-of-function approaches were used to investigate the underlying mechanisms.

**Results:**

MHE reduced lipid accumulation and oxidative stress in palmitic acid-treated hepatocytes and suppressed TGF-β-induced hepatic stellate cell activation, fibrogenic gene expression, and SMAD2/3 phosphorylation in HSC-LX2 cells. In CDAHFD-fed mice, oral administration of MHE attenuated hepatic steatosis, inflammatory injury, and collagen deposition. Mechanistically, MHE and melandryoside restored PPM1A-SMAD interaction and reduced SMAD2/3 phosphorylation, whereas PPM1A knockdown diminished their anti-fibrotic effects.

**Conclusion:**

MHE and melandryoside alleviated pathological features of MASH by reducing metabolic stress and suppressing fibrotic signaling. These findings identify melandryoside as a bioactive constituent of Melandrii Herba and suggest that regulation of PPM1A-SMAD signaling may represent a potential therapeutic strategy for MASH.

## 1 Introduction

Metabolic dysfunction-associated steatotic liver disease has emerged as the most prevalent chronic liver disease worldwide, paralleling the rising prevalence of obesity and type 2 diabetes mellitus (Miao et al., 2024). Although simple steatosis is generally benign, a substantial proportion of patients progress to metabolic dysfunction-associated steatohepatitis (MASH), an aggressive phenotype characterized by hepatocellular lipotoxicity, ballooning injury, chronic inflammation, and progressive fibrosis (Wang and Friedman, 2023). While the initiating insult in MASH is metabolic dysregulation driven by lipid overload, long-term clinical outcomes, including liver-related mortality and the need for transplantation, are most strongly determined by fibrosis stage (Zhu and Cai, 2025). The transition of quiescent hepatic stellate cells (HSCs) into proliferative, extracellular matrix-producing myofibroblasts is a pivotal event in fibrosis progression (Horn and Tacke, 2024). Despite recent approval of the thyroid hormone receptor-β agonist resmetirom, therapeutic options that effectively arrest or reverse fibrosis remain limited, and treatment responses are often incomplete (Zhu and Cai, 2025). Therefore, additional mechanistically distinct therapeutic strategies that concurrently target upstream metabolic injury and downstream fibrogenic signaling are needed.

HSC activation is orchestrated by a complex network of profibrotic mediators, among which transforming growth factor-β (TGF-β) is a central regulator (Meng et al., 2016; Li et al., 2022). Upon ligand binding, TGF-β receptors induce phosphorylation of receptor-regulated SMADs (SMAD2 and SMAD3), which form complexes with SMAD4 and translocate to the nucleus to promote transcription of fibrogenic genes such as α-smooth muscle actin (ACTA2) and collagen type I (COL1A1) (Meng et al., 2016). Under physiological conditions, this pathway is tightly constrained by endogenous negative-feedback mechanisms that limit excessive tissue scarring. Protein phosphatase magnesium-dependent 1A (PPM1A; also known as PP2Cα) is a serine/threonine phosphatase that directly dephosphorylates nuclear SMAD2/3, thereby terminating canonical TGF-β signaling and facilitating SMAD recycling to the cytoplasm (Lin et al., 2006). Impaired resolution of this checkpoint has been implicated in persistent SMAD activation during chronic fibrotic injury (Matsuzaki, 2012). Thus, restoration of PPM1A function or reinforcement of PPM1A-dependent SMAD signal termination may represent a mechanistically distinct anti-fibrotic strategy.

Natural products, owing to their structural diversity and multi-target pharmacological properties, are increasingly recognized as valuable sources of therapeutics for multifactorial metabolic diseases such as MASH (Chen et al., 2025; Wang et al., 2023). In particular, plant-derived polyphenols and related phytochemicals have been shown to ameliorate fatty liver disease through coordinated regulation of lipid metabolism, signaling pathways, and the gut microbiota (Li et al., 2025), and dietary natural products such as Cornus officinalis vinegar can reduce hepatic lipid droplet accumulation and steatosis in fatty liver disease models (Cao et al., 2024). Melandrii Herba, derived from the roots of *Melandryum firmum* (Siebold & Zucc.) Rohrb is a traditional medicinal herb used in Korea, China, and Japan. In classical East Asian medicine, including the *Donguibogam*, it has been prescribed to promote blood circulation, relieve blood stasis, and alleviate inflammatory or swelling-related disorders (Heo, 2009). Recent pharmacological studies further suggest relevance of Melandrii Herba to the metabolic–fibrotic axis of MASH, as extracts have been reported to improve obesity-associated lipid abnormalities in high-fat diet models and suppress inflammatory signaling in macrophages (Jeong et al., 2016; Kim et al., 2020). In addition, extracts of Melandrii Herba have been shown to attenuate oxidative stress-associated cellular injury, supporting potential cytoprotective activity under metabolic stress conditions (Lee et al., 2017). Phytochemical investigations have identified multiple constituents within *M. firmum*, including sterol- and triterpenoid-related compounds, further supporting the presence of bioactive principles in the extract (Zheng et al., 2008; Zhang et al., 2015). However, whether Melandrii Herba directly ameliorates MASH-associated liver injury and fibrosis, and which constituent(s) mediate such effects, remain unclear.

In the present study, we investigated the hepatoprotective and anti-fibrotic effects of Melandrii Herba ethanol extract (MHE) and its purified constituent melandryoside using *in vitro* and *in vivo* models relevant to MASH. We hypothesized that MHE and melandryoside would alleviate disease progression through two complementary pathological nodes: mitigation of hepatocellular lipotoxicity and oxidative stress, and direct suppression of TGF-β-driven HSC activation. Mechanistically, these two nodes are not independent but are sequentially linked: hepatocellular lipid overload generates lipotoxic and oxidative stress that promotes inflammatory signaling and paracrine activation of hepatic stellate cells, which in turn amplifies canonical TGF-β–SMAD signaling to drive fibrosis. Because reactive oxygen species and TGF-β signaling reinforce one another in a feed-forward manner, interventions that simultaneously dampen upstream metabolic injury and reinforce downstream signal termination would be expected to interrupt this self-amplifying cascade at two coupled points. Using cultured hepatic cells and a choline-deficient, L-amino acid-defined, high-fat diet (CDAHFD)-induced mouse model of MASH, we evaluated whether MHE attenuates steatosis, inflammation, and fibrosis. We further examined whether the anti-fibrotic effects of MHE and melandryoside depend on restoration of a phosphatase-mediated SMAD termination mechanism centered on PPM1A. This study identifies melandryoside as a bioactive constituent of MHE and implicates PPM1A-dependent SMAD signal termination as a potential therapeutic mechanism in MASH.

## 2 Materials and methods

### 2.1 General experimental procedures

NMR spectra, including proton experiment, were acquired on JEOL JNM-ECZ400S (400 MHz for ^1^H, 100 MHz for ^13^C) equipped with a 5 mm TCI CryoProbe. Chemical shifts are reported in ppm (δ). Preparative high-performance liquid chromatography (HPLC) was performed with a Waters 1525 binary HPLC pump equipped with a Waters 996 photodiode array detector (Waters Corporation, Milford, CT, United States). Semi-preparative HPLC was conducted using an Agilent 1100 series HPLC System with HPLC UV-Vis Detectors (Agilent Technologies, Santa Clara, CA, United States). LC/MS analysis was conducted on an Agilent 1260 Infinity II Series HPLC system (Agilent Technologies, Santa Clara, CA, United States) equipped with a diode array detector and a 6130 Series ESI mass spectrometer using an analytical Agilent column (3.0 × 150 mm, 3.5 μm). Medium-pressure liquid chromatography (MPLC) was performed using a CombiFlash Rf system equipped with a RediSep® Rf C18 reversed-phase column (Teledyne ISCO, United States). Silica gel 60 (Merck; 230–400 mesh) and RP-C18 silica gel (Merck; 230–400 mesh) were used for column chromatography. Merck precoated silica gel F254 plates and RP-18 F254s plates were used for thin-layer chromatography (TLC). TLC detected spots using UV light or heating after spraying with anisaldehyde-sulfuric acid. Three-dimensional molecular modeling was performed using ChemDraw Ultra.

### 2.2 Cell culture

HSC-LX2 cells (Merck Millipore, NJ; Cat# SCC064) were maintained with Dulbecco’s Modified Eagle’s Medium (DMEM) containing 2% fetal bovine serum (FBS; Gibco, BRL, Grand Island, NY) and 1% penicillin/streptomycin (ThermoFisher Scientific, Waltham, MA) at 37 °C and 5% CO_2_. Then cells were co-treated with TGF-β (10 ng/mL; Gibco, BRL, Grand Island, NY; Cat# 100-21-100UG) and MHE (25, 50, and 100 μg/mL), melandryoside (1, 3, and 10 µM), or vehicle (DMSO). Cells were then harvested for qPCR or immunoblotting. Short pretreatment (2 h) with MHE or melandryoside followed by brief TGF-β exposure (30 min) was used for measuring SMAD2/3 phosphorylation. Prolonged TGF-β stimulation (24 h) was used for experiments assessing fibrotic protein and gene expression using Western blot and qPCR analysis.

HepG2 cells were purchased from American Type Culture Collection (ATCC, Manassas, VA) and cultured in DMEM containing 10% FBS and 1% penicillin/streptomycin. FL83B cells, a non-transformed mouse hepatocyte-derived liver cell line, purchased from the American Type Culture Collection (ATCC, Manassas, VA), were maintained in F-12K medium supplemented with 10% FBS and 1% penicillin/streptomycin. For intracellular lipid accumulation, cells were cultured in a medium with the addition of 400 μmol/L palmitic acid for 24 h and then cells were harvested for further analysis. Images of cells stained with Oil Red O were obtained with EVOS FL (Thermo Fisher Scientific). For quantification, Oil Red O was extracted with isopropanol and absorbance was measured at 520 nm.

### 2.3 *In vitro* cytotoxicity test

To analyze the cytotoxic effect of MHE on HSC-LX2 cells or HepG2 cells, we performed the MTT (3-(4,5-dimethylthiazol-2-yl)-2,5-diphenyl tetrazolium bromide) assay which is previously elucidated (van Meerloo et al., 2011).

### 2.4 Immunoblotting and immunoprecipitation

Supernatants containing protein contents were measured by Bradford Protein Assay (Bio-Rad Laboratories, Hercules, CA). Proteins were immunoblotted with anti-ACTA2 (19245, Cell Signaling Technology, Danvers, MA), anti-COL1A1 (72026, Cell Signaling Technology, Danvers, MA), anti-Fibronectin (sc271098, Santa Cruz biotechnology, Dallas, TX), anti-SMAD2/3 (3102, Cell Signaling Technology, Danvers, MA), anti-phospho-SMAD2 (3108, Cell Signaling Technology, Danvers, MA), anti-phospho-SMAD3 (9520, Cell Signaling Technology, Danvers, MA), anti-PPM1A (sc166662, Santa Cruz biotechnology, Dallas, TX), anti-NRF2 (12721, Cell Signaling Technology, Danvers, MA), anti-HSP90 (4877, Cell signaling Technology, Danvers, MA), anti-Tubulin (2146, Cell signaling Technology, Danvers, MA), and anti-Lamin B1 (sc-374015, Santa Cruz biotechnology, Dallas, TX). For immunoprecipitation, cleared cell extracts were incubated with specific antibodies overnight. Then, the protein-antibody samples were mixed with protein A/G agarose beads for 2 h and precipitated.

Densitometric analysis was performed using ImageJ (U.S. National Institutes of Health, Bethesda, MD, United States). For densitometric analyses, phosphorylated SMAD2 and SMAD3 were normalized to their respective total protein levels, whereas other quantified proteins were normalized to the corresponding loading control (HSP90, Tubulin, or Lamin B1).

### 2.5 Extraction of nuclear and cytoplasmic proteins

Cells were lysed in ice-cold fractionation buffer (20 mM HEPES, pH 7.4, 10 mM KCl, 2 mM MgCl_2_, 1 mM EDTA, 1 mM EGTA, 1 mM DTT, and protease inhibitor cocktail) and mechanically disrupted by repeated passage through a 27-gauge needle. Following differential centrifugation (720 × g), the supernatant was collected as the cytoplasmic fraction, whereas the nuclear pellet was washed, resuspended in TBS containing 0.1% SDS, and briefly sonicated. Both cytoplasmic and nuclear fractions were subjected to immunoblot analysis.

### 2.6 Quantitative PCR

Total RNA was isolated using a TRIzol reagent (Thermo Fisher Scientific, Waltham, MA). RNA reverse transcription was performed using an ABI Reverse Transcription Kit (Thermo Fisher Scientific, Waltham, MA). Quantitative PCR was performed with a CFX Duet Real-Time PCR system (Bio-Rad, Hercules, CA) following the manufacturer’s instructions. Relative mRNA expression levels of each gene were normalized to the expression level of the TATA-binding protein TBP. Primer sequences are listed in Table 1.

**TABLE 1:** Primers.

Gene name	Primer sequences for qPCR	
Human gene	Forward primer	Reverse primer
*ACTA2*	AAT​GCA​GAA​GGA​GAT​CAC​GG	TCC​TGT​TTG​CTG​ATC​CAC​ATC
*COL1A1*	CCC​CTG​GAA​AGA​ATG​GAG​ATG	TCC​AAA​CCA​CTG​AAA​CCT​CTG
*FN*	ACT​GTA​CAT​GCT​TCG​GTC​AG	AGT​CTC​TGA​ATC​CTG​GCA​TTG
*SREBF1*	CAA​CAC​AGC​AAC​CAG​AAA​CTC	CTC​CAC​CTC​AGT​CTT​CAC​G
*FASN*	CAA​GCT​GAA​GGA​CCT​GTC​TAG	CGG​AGT​GAA​TCT​GGG​TTG​ATG
*SCD1*	AGT​TCT​ACA​CCT​GGC​TTT​GG	GTT​GGC​AAT​GAT​CAG​AAA​GAG​C
*KEAP1*	CTG​GAG​GAT​CAT​ACC​AAG​CAG​G	GGA​TAC​CCT​CAA​TGG​ACA​CCA​C
*NRF2*	TCA​GCG​ACG​GAA​AGA​GTA​TGA	CCA​CTG​GTT​TCT​GAC​TGG​ATG​T
*HMOX1*	CCA​GGC​AGA​GAA​TGC​TGA​GTT​C	AAG​ACT​GGG​CTC​TCC​TTG​TTG​C
*PPM1A*	ACC​CAA​AGT​ATC​GCC​AGA​AG	GGG​ATG​TTC​TCA​CTC​GCT​AAT
*TBP*	CCA​CTC​ACA​GAC​TCT​CAC​AAC	CTG​CGG​TAC​AAT​CCC​AGA​ACT

Mouse gene	Forward primer	Reverse primer
*Acta2*	GTG​AAG​AGG​AAG​ACA​GCA​CAG	GCC​CAT​TCC​AAC​CAT​TAC​TCC
*Col1a1*	CAT​AAA​GGG​TCA​TCG​TGG​CT	TTG​AGT​CCG​TCT​TTG​CCA​G
*Fn*	CTT​TGG​CAG​TGG​TCA​TTT​CAG	ATT​CTC​CCT​TTC​CAT​TCC​CG
*Tnf*	CCC​TCA​CAC​TCA​GAT​CAT​CTT​CT	GCT​ACG​ACG​TGG​GCT​ACA​G
*Ccl2*	TTA​AAA​ACC​TGG​ATC​GGA​ACC​AA	GTT​CAC​CGT​AAG​CCC​AAT​TT
*Il1b*	GCA​CTA​CAG​GCT​CCG​AGA​TGA​AC	TTG​TCG​TTG​CTT​GGT​TCT​CCT​TGT
*Tbp*	ACC​CTT​CAC​CAA​TGA​CTC​CTA​TG	TGA​CTG​CAG​CAA​ATC​GCT​TGG

### 2.7 ROS analysis

Intracellular ROS levels were assessed in HepG2 and HSC-LX2 cells using the DCFH-DA assay kit (ab113851, Abcam, Cambridge, MA) following the manufacturer’s instructions. Briefly, cells (2 × 10^5^ per well) were serum starved for 6 h and then treated with PA (400 µM) with or without MHE or melandryoside at the indicated concentrations for 24 h. After incubation, cells were washed with 1× buffer and treated with DCFH-DA (25 µM) for 45 min at 37 °C in the dark. Fluorescence was measured at Ex/Em = 485/535 nm using a microplate reader to quantify intracellular ROS levels.

### 2.8 Expression plasmids and RNA interference

WT human PPM1A was obtained from Korea Research Institute of Bioscience and Biotechnology (KRIBB). PPM1A mutants (R174G and D239N) were generated using QuickChange II Site-Directed Mutagenesis Kit obtained from Agilent Technologies (200524, Santa Clara, CA, United States). All mammalian expression plasmids were transfected using Lipofectamine™ 2000 transfection reagent (11668019, ThermoFisher Scientific, Waltham, MA) following the manufacturer’s instructions. An siRNA targeting human PPM1A (5′- CGCCAGAAGCAGUGA AGA A-3′) was purchased from Shanghai GenePharma (Shanghai, China). HSC-LX2 cells were transfected with siRNAs using Lipofectamine™ RNAiMAX transfection reagent (13778150, ThermoFisher Scientific, Waltham, MA).

### 2.9 Mice

All animal experiments were performed according to procedures approved by the Ulsan National Institute of Science and Technology’s Institutional Animal Care and Use Committee (UNISTIACUC-25-022). Mice were maintained in a specific pathogen–free animal facility under a 12-h light/dark cycle at a temperature of 21 °C and allowed free access to water and food. Seven-week-old male C57BL/6J mice (DBL, Chungbuk, Republic of Korea) were fed a CDAHFD (L-amino acid diet with 60 kcal% fat with 0.1% methionine and no added choline, A06071302, Research Diets Inc., New Brunswick, NJ) for 12 weeks. To model a therapeutic (interventional) rather than a preventive paradigm, MHE administration was initiated after disease establishment. Mice were fed CDAHFD for 8 weeks to induce steatohepatitis and early fibrosis, after which MHE (25 or 50 mg/kg) or vehicle was administered once daily by oral gavage from week eight to week 12 (the final 4 weeks), with CDAHFD feeding continued throughout the treatment period. Animals were sacrificed at week 12.

### 2.10 Biochemical analysis

Body weight was monitored at regular intervals throughout the study. To analyze metabolic parameters, serum ALT (700260, Cayman Chemical, Ann Arbor, MI), AST (701640, Cayman Chemical, Ann Arbor, MI), TG (10010303, Cayman Chemical, Ann Arbor, MI), and cholesterol (BM-CHO-100, BioMax, Republic of Korea) were performed according to the manufacturer’s instructions.

### 2.11 Histological analysis

Mouse liver tissues were harvested and subsequently fixed with 4% formalin. Liver sections were stained with H&E and Oil Red O staining to evaluate lipid accumulation. Mayer’s hematoxylin was used as a counterstain for every slide. Liver fibrosis was further assessed by Sirius red staining. Images were obtained with the CELENA® X High Content Imaging System (Logos Biosystems, Gyeonggi-do, Republic of Korea). Histological scoring was performed according to the NASH Clinical Research Network (NASH CRN) scoring system (Kleiner et al., 2005; Brunt et al., 2011). Fibrosis was staged on a scale of 0–4 by three independent investigators who were blinded to the treatment groups. Inter-rater agreement was evaluated by comparing the independently assigned fibrosis scores, and any discrepant scores were resolved by consensus to obtain the final fibrosis score. Fibrosis scores are presented as individual values with group means.

### 2.12 Interactome and pathway analysis

Protein–protein interaction data for SMAD2 and SMAD3 were obtained from public interactome resources, including Wiki-Pi, IntAct, and STRING. After integrating the three datasets, proteins present in both SMAD2-and SMAD3-associated interaction networks were identified and used for subsequent analysis.

The overlapping protein set was analyzed using Reactome pathway enrichment to determine significantly associated signaling pathways. A network of the shared proteins was constructed using STRING interaction data to define their connectivity, which enabled the identification of regulatory components within the SMAD-associated interaction network, including phosphatase and other modulatory proteins.

### 2.13 Extraction and isolation

Dried leaves of *M. firmum* (1 kg), belonging to the family Caryophyllaceae, were obtained in January 2024 from Kwangmyungdang Pharmaceutical (Yeongcheon, Gyeongsangbuk-do, Republic of Korea), and were authenticated by one of the authors (S.R.L.). A voucher specimen (PNU-PL-2025-15) of the plant is stored at the herbarium of the College of Pharmacy, Pusan National University, Busan, Korea. The dried plant material was extracted three times with 70% EtOH at room temperature. The combined extracts were concentrated *in vacuo* to afford a crude dark green 70% EtOH extract (71 g, 7.1% yield). The 70% EtOH fraction (71 g) was subjected onto silica gel column chromatography (230–400 mesh) and fractionated by a gradient solvent system of CH_2_Cl_2_-MeOH (100:1 to 0:1, v/v) to obtain two fractions (E.1-E.2). Fraction.E.2 (52.0 g) was loaded on a Diaion HP-20 chromatography column eluted with MeOH-H_2_O (4:1 to 1:0, v/v) to give four fractions (E.2.1-E.2.4). Fraction.E.2.3 (25.0 g) was separated by silica gel column chromatography with a gradient solvent system of CH_2_Cl_2_-MeOH (20:1 to 0:1, v/v) to obtain five fractions (E.2.3.1-E.2.3.5). Fraction E.2.3.4 (15.0 g) was subjected to passage over an RP-C18 silica gel column (230–400 mesh) with gradient system of MeOH-H_2_O (3:7 to 1:0, v/v) to obtain five fractions (E.2.3.4.1-E.2.3.4.5). Fraction.E.2.3.4.1 (12.0 g) was subsequently separated by silica gel column chromatography with a gradient solvent system of CH_2_Cl_2_-MeOH (10:1 to 0:1, v/v) to obtain four fractions (E.2.3.4.1.1-E.2.3.4.1.4). Fraction.E.2.3.4.1.3 (2.72 g) was separated using preparative reverse-phase HPLC (YMC C18, 250 × 21.2 mm, 5 μm, at a flow rate of 5 mL/min) with gradient solvent system of CH_3_CN-H_2_O (1:9 to 4.5:5.5, v/v) to give four fractions (E.2.3.4.1.3.1-E.2.3.4.1.3.4). Subfraction.E.2.3.4.1.3.2 (450 mg) was isolated using semi-preparative reverse-phase HPLC (YMC C18, 250 × 21.2 mm, 5 μm, at a flow rate of 2 mL/min) using an isocratic elution of 35% aqueous MeOH to afford compound 2 (14.5 mg, *t*_R_ = 56 min), 3 (7.5 mg, *t*_R_ = 60 min), 1 (5.2 mg, *t*_R_ = 65 min), 4 (2.1 mg, *t*_R_ = 69 min). Fraction.E.1 (6.6 g) was separated by silica gel column chromatography with a gradient solvent system of CH_2_Cl_2_-MeOH (100:1 to 0:1, v/v) to obtain six fractions (E.1.1-E.1.6). Fraction.E.1.5 (1.0 g) was separated using medium pressure liquid chromatography (MPLC; RediSep® Rf C18, 12 g, at a flow rate of 13 mL/min) with a gradient solvent system of MeOH-H_2_O (1:9 to 1:0, v/v) to afford six fractions (E.1.5.1- E.1.5.6). Fraction.E.1.5.3 (110.2 mg) was separated using preparative reverse-phase HPLC (YMC C18, 250 × 21.2 mm, 5 μm, at a flow rate of 5 mL/min) with gradient solvent system of CH_3_CN-H_2_O (1:9 to 5:5, v/v) to give five fractions (E.1.5.3.1-E.1.5.3.5). Subfraction.E.1.5.3.5 (16.3 mg) was isolated using semi-preparative reverse-phase HPLC (YMC C18, 250 × 21.2 mm, 5 μm, at a flow rate of 2 mL/min) using an isocratic elution of 51% aqueous MeOH to afford compound 5 (1.3 mg, *t*_R_ = 75 min). Fraction.E.1.2 (3.2 g) was separated using medium pressure liquid chromatography (MPLC; RediSep® Rf C18, 12 g, at a flow rate of 13 mL/min) with a gradient solvent system of MeOH-H_2_O (3:7 to 1:0, v/v) to afford three fractions (E.1.2.1- E.1.2.3). Fraction.E.1.2.1 (140.3 mg) was separated using preparative reverse-phase HPLC (YMC C18, 250 × 21.2 mm, 5 μm, at a flow rate of 5 mL/min) with gradient solvent system of CH_3_CN-H_2_O (1:9 to 5:5, v/v) to give five fractions (E.1.2.1.1-E.1.2.1.5). Subfraction.E.1.2.1.2 (13.0 mg) was isolated using semi-preparative reverse-phase HPLC (YMC C18, 250 × 21.2 mm, 5 μm, at a flow rate of 2 mL/min) using an isocratic elution of 58% aqueous MeOH to afford compound 6 (2.7 mg, *t*_R_ = 40 min). Subfraction.E.1.2.1.3 (18.3 mg) was isolated using semi-preparative reverse-phase HPLC (YMC C18, 250 × 21.2 mm, 5 μm, at a flow rate of 2 mL/min) using an isocratic elution of 34% aqueous MeOH to afford compound 7 (0.6 mg, *t*_R_ = 42 min).

*Melandryoside* (1): Yellow amorphous powder; [α]-12.5 (*c* 0.5, MeOH); UV (MeOH) *λ*_max_ (log ε) 270 (3.24), 338 (3.01); ^1^H-NMR (400 MHz) and ^13^C-NMR (100 MHz) NMR data (Supplementary Figures S2–S5), see Table 2; HR-ESIMS (positive-ion mode) *m/z* 595.1654 [M + H]^+^ (calc. for C_27_H_31_O_15_, 595.1657) (Supplementary Figure S1).

**TABLE 2:** ^1^H and^13^C NMR data of melandryoside (1) in CD_3_OD.^a^ ^,^ ^b^.

Position	1	
	*δ*_C_	*δ*_H_ (*J* in Hz)
2	164.6 s	​
3	102.7 d	6.65 s
4	182.5 s	​
5	160.5 s	​
6	107.7 s	​
7	163.5 s	​
8	94.1 d	6.52 s
9	157.2 s	​
10	103.7 s	​
1′	122.0 s	​
2′	109.1 d	7.49 d (2.0)
3′	148.0 s	​
4′	150.8 s	​
5′	115.3 d	6.94 d (8.5)
6′	120.4 d	7.51 d (8.5)
3′-OCH_3_	55.1 q	3.97 s
1″	72.1 d	4.95 d (10.0)
2″	81.2 d	3.94 m
3″	78.8 d	3.64 m
4″	72.2 d	3.66 m
5″	81.8 d	3.42 m
6″	61.2 t	3.75 m
​	​	3.88 m
1‴	108.4 d	5.21 d (1.5)
2‴	76.3 t	3.71 m
3‴	81.1 s	​
4‴	76.4 d	2.97 m
​	​	3.70 m
5‴	60.3 t	3.27 m
​	​	3.29 m

^*a*^ Coupling constants (in parentheses) are shown in Hz.

^*b*^ 13C NMR, data were assigned based on HSQC, and HMBC, experiments.

The purity of the isolated melandryoside was confirmed by HPLC (UV detection at 254 nm; Supplementary Figure S1). All cell-based experiments were performed using melandryoside obtained from a single isolation batch, which was stored at −80 °C until use to minimize chemical degradation.

### 2.14 Acid hydrolysis of melandryoside

Melandryoside (1, 0.5 mg) was dissolved in a mixture of H_2_O (0.25 mL) and 1 N HCl (0.25 mL) in a sealed screw-cap vial and heated at 80 °C for 1 h. After cooling to room temperature, the reaction mixture was diluted with H_2_O (0.3 mL) and extracted with EtOAc (1.0 mL × 2). The combined EtOAc layers were concentrated *in vacuo* to afford the aglycone fraction, whereas the aqueous layer containing the liberated sugars was concentrated under reduced pressure for further analysis (Lee et al., 2021; Sharaf et al., 2017).

### 2.15 Statistical analysis

All data are presented as the mean ± SEM. Statistical analysis were performed using GraphPad Prism 9.3.1 (GraphPad Software, La Jolla, CA, United States). Two-tailed Student’s t-tests were used for two-group comparisons, and one-way ANOVA followed by Tukey’s *post hoc* test was used for multiple-group comparisons. *P* values are indicated as * for *p* < 0.05, ** for *p* < 0.01, and *** for *p* < 0.001.

## 3 Results

### 3.1 MHE suppresses TGF-β–driven HSC activation and attenuates SMAD2/3 phosphorylation

**FIGURE 1:**
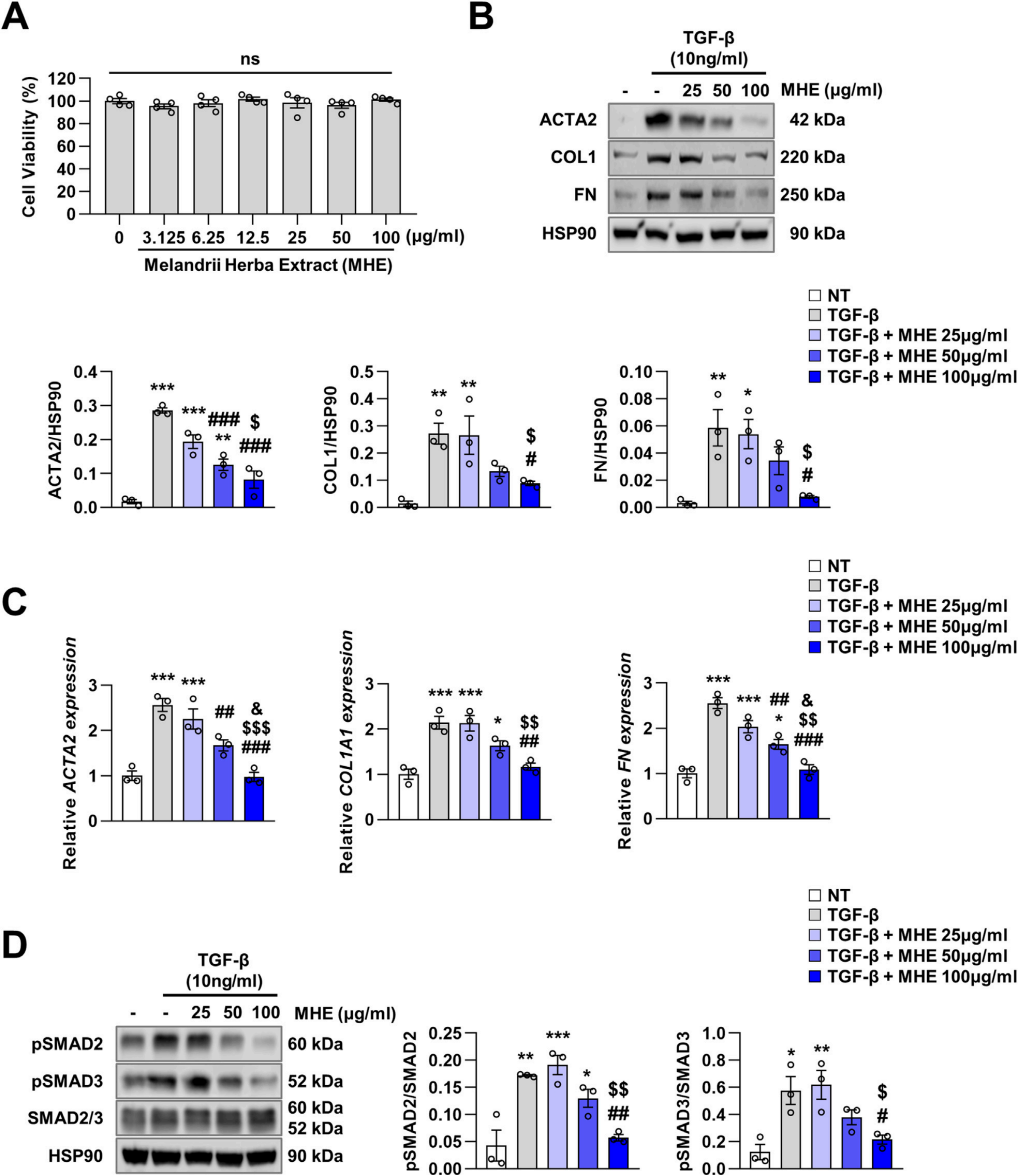
MHE regulates TGF-β–stimulated fibrotic responses in HSC-LX2 cells. **(A)** Viability of HSC-LX2 cells treated with MHE. **(B)** Immunoblot and quantitative analysis of ACTA2, COL1, and FN expression. **(C)** Relative mRNA expression levels of *ACTA2*, *COL1A1*, and *FN*. **(D)** Immunoblot and quantitative analysis of key proteins of the SMAD signaling pathway. Data are presented as mean ± SEM (n = 3 per group). One-way ANOVA followed by Tukey multiple comparison test. *p < 0.05, **p < 0.01, ***p < 0.001 vs. NT; ^##^ *p <* 0.01, ^###^ *p <* 0.001 vs. TGF-β; ^$^ *p <* 0.05, ^$$^ *p <* 0.01, ^$$$^ *p <* 0.001 vs. TGF-β + MHE 25 μg/mL; ^&^ *p <* 0.05 vs. TGF-β + MHE 50 μg/mL.

To assess whether MHE modulates the fibrogenic activation program of HSCs, HSC-LX2 cells were stimulated with TGF-β and evaluated using phenotypic and signaling readouts under conditions optimized for either downstream gene induction or proximal SMAD activation. First, we established non-cytotoxic working concentrations for mechanistic experiments. Treatment of HSC-LX2 cells with increasing doses of MHE did not affect cell viability, indicating that subsequent changes in fibrotic markers were not secondary to overt cytotoxicity (Figure 1A). We next examined whether MHE modulates the fibrogenic response elicited by prolonged TGF-β exposure. Immunoblotting revealed that TGF-β robustly induced canonical markers of HSC activation, including ACTA2 (α-SMA), COL1, and fibronectin (FN). Quantitative analysis further confirmed that co-treatment with MHE reduced the abundance of these proteins in a dose-dependent manner (Figure 1B). Consistent with these protein-level effects, MHE decreased the mRNA expression of fibrogenic marker genes under the same conditions (Figure 1C), providing convergent evidence that MHE suppresses TGF-β–driven profibrotic transcriptional outputs in HSC-LX2 cells.

Because canonical TGF-β signaling is initiated by phosphorylation of receptor-regulated SMADs (Meng et al., 2016), we next investigated whether MHE attenuates early SMAD2/3 activation. HSC-LX2 cells were pretreated with MHE for 2 h and subsequently challenged with TGF-β for 30 min, conditions optimized to capture proximal signaling events. Under these conditions, MHE attenuated phosphorylation of both SMAD2 and SMAD3 compared with TGF-β alone, as confirmed by quantitative analysis (Figure 1D). Together, these results link suppression of fibrogenic marker induction with reduced canonical SMAD2/3 activation, supporting a role for MHE in limiting TGF-β–driven HSC activation.

### 3.2 MHE attenuates palmitate-induced lipotoxicity and oxidative stress in hepatocytes and stellate cells

Having established that MHE suppresses TGF-β–driven fibrogenic activation in hepatic stellate cells (Figure 1), we next examined the upstream hepatocellular events that drive this response. Hepatocyte lipotoxic injury caused by excessive fatty acid loading is a major upstream driver of hepatic inflammation and can promote stellate cell activation through reactive oxygen species (ROS)-dependent signaling (Horn and Tacke, 2024). To examine whether MHE modulates this pathogenic input, we established a palmitic acid (PA)-induced lipotoxicity model in HepG2 hepatocytes and assessed lipid accumulation, lipogenic gene programs, and oxidative stress readouts.

**FIGURE 2:**
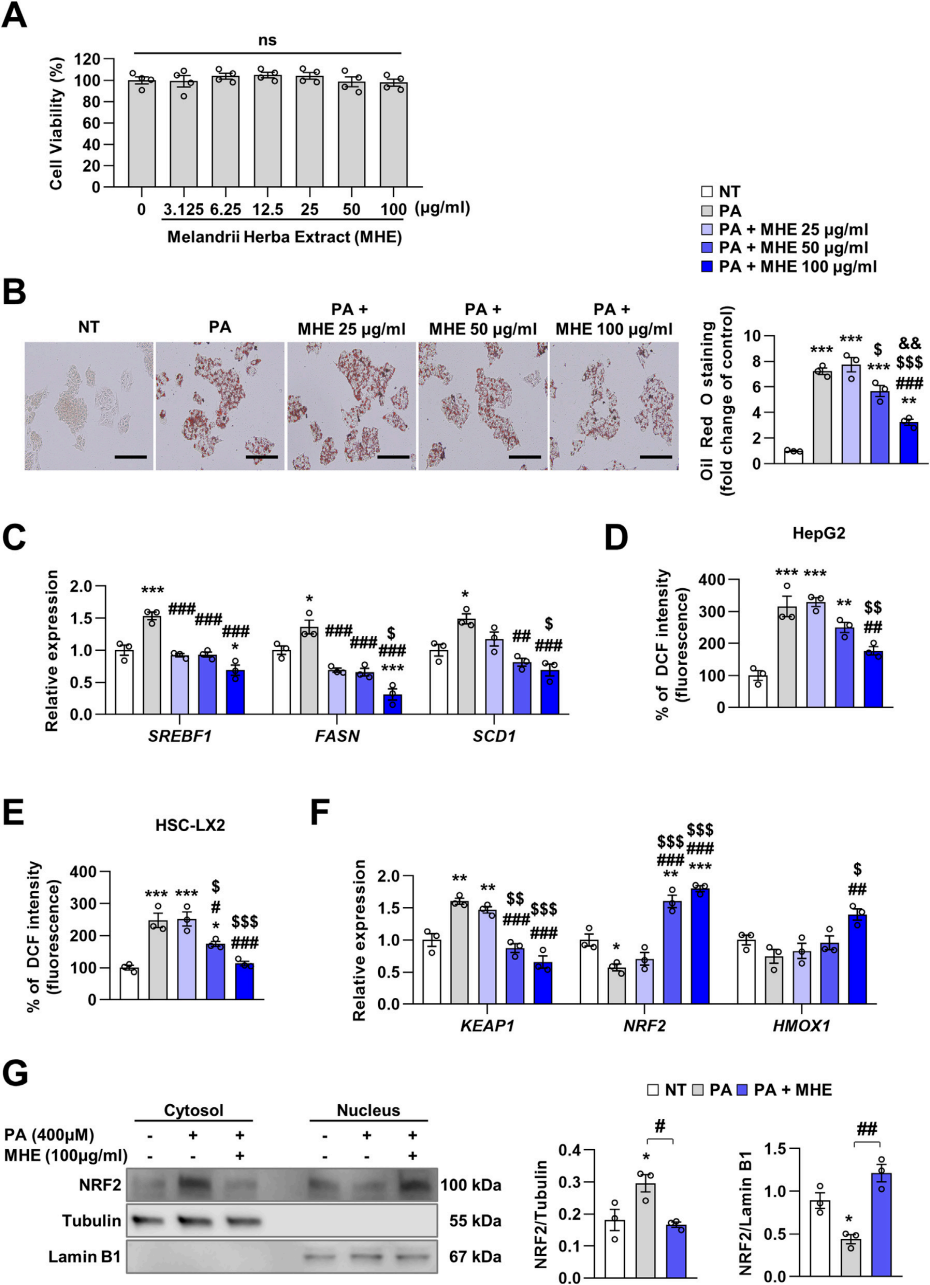
MHE ameliorates palmitic acid-induced lipid accumulation and oxidative stress in hepatocytes. **(A)** Viability of HepG2 cells treated with MHE. **(B)** Representative Oil Red O staining and quantification of intracellular lipid accumulation in HepG2 cells (scale bar, 100 μm). **(C)** Relative mRNA expression of *de novo* lipogenesis-related genes in HepG2 cells. **(D)** Intracellular ROS levels in HepG2 cells. **(E)** Intracellular ROS levels in HSC-LX2 cells. **(F)** Relative mRNA expression of oxidative stress-related genes in HepG2 cells. **(G)** Immunoblot and quantitative analysis of nuclear and cytoplasmic NRF2 expression in HepG2 cells. Data are presented as mean ± SEM (n = 3 per group). One-way ANOVA followed by Tukey multiple comparison test. *p < 0.05, **p < 0.01, ***p < 0.001 vs. NT; ^#^ *p <* 0.05, ^##^ *p <* 0.01, ^###^ *p <* 0.001 vs. PA; ^$^ *p <* 0.05, ^$$^ *p <* 0.01, ^$$$^ *p <* 0.001 vs. PA + MHE 25 μg/mL; ^&&^ *p <* 0.01 vs. PA + MHE 50 μg/mL.

MHE did not affect HepG2 viability across the concentrations used for mechanistic analysis (Figure 2A), indicating that subsequent changes were not attributable to overt cytotoxicity. Under PA challenge, HepG2 and FL83B cells exhibited a marked increase in intracellular lipid droplet accumulation, whereas co-treatment with MHE reduced Oil Red O-positive lipid deposition. Quantification confirmed a dose-dependent decrease in lipid accumulation in both cell lines (Figure 2B; Supplementary Figure S6).

To determine whether the reduction in lipid burden was accompanied by modulation of lipogenic transcriptional programs, we quantified genes involved in *de novo* lipogenesis. MHE decreased the mRNA expression of lipogenesis-associated genes, including *SREBF1*, *FASN*, and *SCD1* (Figure 2C), consistent with attenuation of PA-driven lipogenic responses rather than a staining-only effect. Because oxidative stress is a key mediator linking hepatocyte lipotoxicity to fibrogenic signaling, we next measured intracellular ROS using a DCFH-DA assay. Following serum starvation, cells were exposed to PA with or without MHE, and ROS were quantified by fluorescence after DCFH-DA loading. MHE reduced ROS accumulation in PA-treated HepG2 cells (Figure 2D). Notably, a similar reduction in ROS was observed in HSC-LX2 cells (Figure 2E), suggesting that MHE also modulates oxidative stress in fibrogenic effector cells. To complement the fluorescence readout, we further examined ROS-associated gene expression in HepG2 cells. MHE decreased *KEAP1* expression while increasing *NRF2* and *HMOX1* transcripts (Figure 2F), supporting broader regulation of oxidative stress-related pathways. To further assess NRF2 activation, nuclear and cytoplasmic fractions were analyzed by immunoblotting. PA increased cytoplasmic NRF2 while reducing nuclear NRF2 accumulation relative to NT cells. In contrast, MHE decreased cytoplasmic NRF2 and increased nuclear NRF2 accumulation relative to PA-treated cells, consistent with restoration of NRF2 nuclear translocation (Figure 2G). Collectively, these results indicate that MHE attenuates PA-induced lipotoxic responses by reducing lipid accumulation and lipogenic gene activation, while limiting ROS-associated stress signaling through promotion of NRF2 nuclear translocation in hepatocytes, with parallel attenuation of oxidative stress in stellate cells. These findings provide a mechanistic link between the upstream hepatoprotective effects of MHE and its downstream anti-fibrotic actions investigated in HSCs.

### 3.3 MHE ameliorates steatosis, inflammatory injury, and fibrosis in a diet-induced mouse model of MASH

**FIGURE 3:**
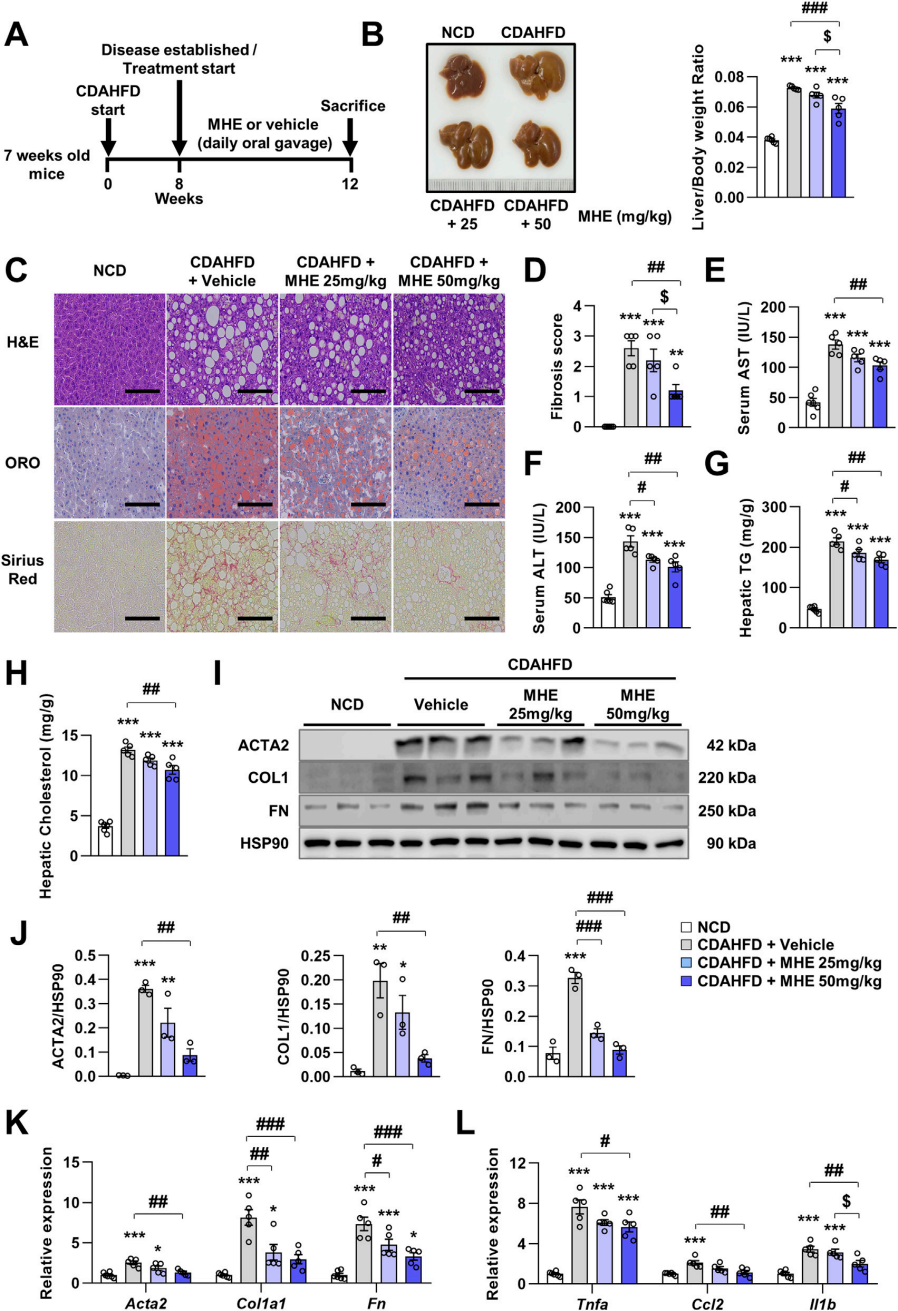
Alterations of hepatic injury, fibrosis, and inflammation following MHE administration in CDAHFD mice. **(A)** Experimental design. Mice were fed CDAHFD for 12 weeks; MHE or vehicle was administered therapeutically from week 8 (after disease establishment) to week 12, with CDAHFD feeding continued throughout. **(B)** Representative liver images and liver-to-body weight ratio. **(C)** Representative images of H&E, ORO staining, and Sirius Red staining of liver sections (scale bar, 100 μm). **(D)** Quantification of hepatic fibrosis score. **(E)** Serum AST levels. **(F)** Serum ALT levels. **(G)** Hepatic TG. **(H)** Hepatic cholesterol. **(I)** Immunoblot analysis of fibrotic markers in liver tissues. **(J)** Quantitative analysis of ACTA2, COL1, and FN normalized to HSP90 protein levels. **(K)** Relative mRNA expression of hepatic fibrosis-related genes. **(L)** Relative mRNA expression of inflammatory cytokines. Data are presented as mean ± SEM [normal control diet (NCD), n = 6; CDAHFD + Vehicle, CDAHFD + MHE 25 mg/kg, and CDAHFD + MHE 50 mg/kg, n = 5 per group]. One-way ANOVA followed by Tukey multiple comparison test. *p < 0.05, **p < 0.01, ***p < 0.001 vs. NCD; ^#^ *p <* 0.05, ^##^ *p <* 0.01, ^###^ *p <* 0.001 vs. CDAHFD + Vehicle; ^$^ *p <* 0.05 vs. CDAHFD + MHE 25 mg/kg.

Because hepatocyte lipotoxicity and oxidative stress represent key upstream drivers of hepatic inflammation and fibrogenic remodeling, we next sought to determine whether the hepatoprotective effects of MHE observed *in vitro* translated into protection against MASH progression *in vivo*. To this end, we performed an *in vivo* proof-of-concept study using a CDAHFD mouse model that recapitulates key histopathological features of MASH, including steatohepatitis and progressive fibrosis. Mice were maintained on CDAHFD for 8 weeks to establish steatohepatitis and early fibrosis, and MHE was then administered therapeutically from week eight to week 12 while dietary challenge was continued, at two doses, enabling assessment of dose responsiveness (Figure 3A). MHE treatment attenuated gross and histological features of hepatic steatosis. Compared with CDAHFD controls, MHE reduced hepatomegaly and decreased the liver-to-body weight ratio in a dose-dependent manner (Figure 3B). H&E− and Oil Red O-stained liver sections showed reduced steatotic changes in MHE-treated mice relative to CDAHFD controls (Figure 3C). Consistent with these histological findings, MHE significantly decreased hepatic triglyceride and total cholesterol contents (Figures 3G,H). These findings indicate that oral MHE attenuates lipid-associated hepatic pathology under sustained dietary challenge.

Importantly, MHE also mitigated fibrosis, a major determinant of disease progression in MASH. Sirius Red staining revealed reduced collagen deposition in MHE-treated animals, accompanied by lower fibrosis scores compared with CDAHFD controls (Figures 3C,D). Furthermore, MHE decreased expression of fibrotic markers, including ACTA2 (α-SMA), COL1A1, and FN, as demonstrated by protein and transcript analyses (Figures 3I–K). Together, these histological and molecular readouts indicate that MHE limits extracellular matrix accumulation and fibrotic remodeling *in vivo* under continued dietary challenge.

Consistent with reduced tissue injury, MHE lowered serum AST and ALT levels relative to CDAHFD controls (Figures 3E,F), supporting attenuation of hepatocellular damage. In parallel, MHE decreased hepatic expression of inflammatory mediators, including *Tnfa*, *Ccl2*, and *Il1b* (Figure 3L), suggesting modulation of the inflammatory milieu associated with fibrogenic progression. Collectively, these *in vivo* findings demonstrate that MHE attenuates steatosis, inflammatory injury, and fibrosis in diet-induced MASH mice when administered therapeutically after disease establishment. These findings are consistent with the hepatoprotective effects of MHE observed *in vitro* and support a mechanistic link between suppression of upstream lipotoxic and oxidative stress signals and attenuation of downstream fibrogenic remodeling.

### 3.4 Network-guided prioritization identified PPM1A as a required node for MHE-dependent regulation of SMAD2/3 signaling

Given that MHE rapidly attenuated TGF-β-induced SMAD2/3 phosphorylation in HSC-LX2 cells (Figure 1D), we reasoned that MHE may engage a regulatory mechanism that constrains canonical SMAD activation. Because SMAD2/3 phosphorylation is dynamically controlled by protein-protein interactions and reversible dephosphorylation (Wrighton et al., 2009), we sought to identify a SMAD-associated regulatory factor that could account for the MHE-mediated suppression of SMAD signaling.

**FIGURE 4:**
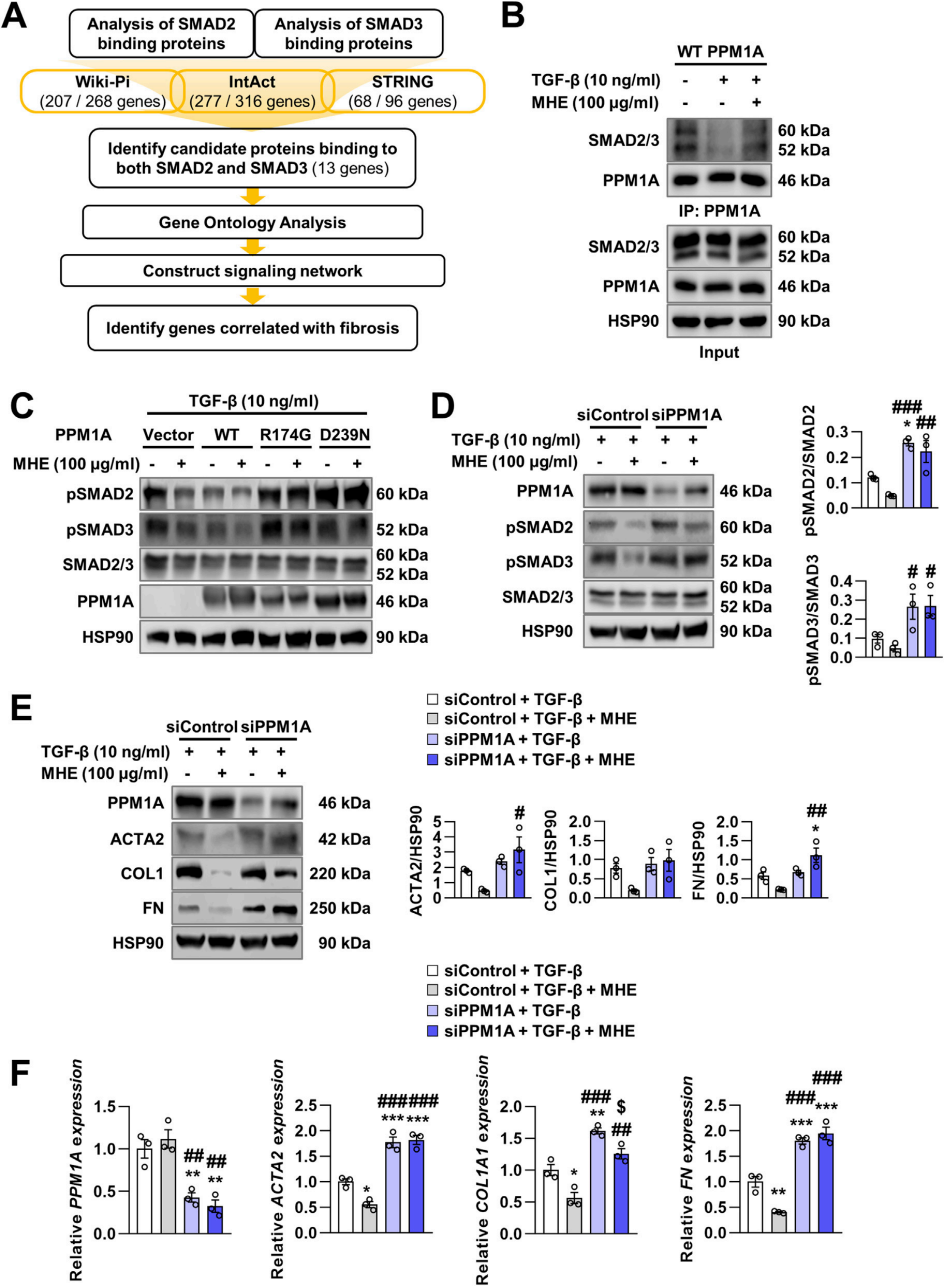
Identification and analysis of PPM1A-SMAD2/3 interactions associated with fibrotic signaling. **(A)** Workflow for bioinformatic identification of SMAD2/3-binding proteins. **(B)** HSC-LX2 cells were transfected with wild-type (WT) PPM1A. The cells were then immunoprecipitated with anti-PPM1A antibody. Precipitates and cell lysates were subjected to immunoblotting for SMAD2/3 and PPM1A. **(C)** Immunoblot analysis of key proteins in the SMAD signaling pathway and PPM1A with WT PPM1A and two catalytically inactive PPM1A mutants (R174G and D239N). **(D,E)** HSC-LX2 cells were administered non-targeting siRNA (siControl) or PPM1A-targeting siRNA (siPPM1A). Immunoblot and quantitative analysis of **(D)** key proteins of the SMAD signaling pathway and **(E)** fibrotic markers and PPM1A. **(F)** Relative mRNA expression levels of PPM1A and hepatic fibrosis-related genes. Data are presented as mean ± SEM (n = 3 per group). One-way ANOVA followed by Tukey multiple comparison test. *p < 0.05, **p < 0.01, ***p < 0.001 vs. TGF-β + siControl; ^#^ *p <* 0.05, ^##^ *p <* 0.01, ^###^ *p <* 0.001 vs. TGF-β + siControl + MHE; ^$^ *p <* 0.05 vs. TGF-β + siPPM1A.

To prioritize candidates in an unbiased manner, we compiled interaction partners of SMAD2 and SMAD3 from independent curated interactome resources (STRING, IntAct, and Wiki-Pi) and focused on proteins shared by both interactomes. This intersection strategy was designed to enrich for core regulators positioned at common checkpoints of canonical signaling rather than SMAD isoform-specific modulators. The integrated analysis yielded 13 overlapping candidates (Figure 4A). We next organized the shared SMAD2/3-interacting proteins by functional class to identify regulators plausibly linked to reduced SMAD phosphorylation. Among these candidates, PPM1A emerged as the only phosphatase within the shared SMAD2/3 set (Supplementary Figure S7). Because PPM1A is a serine/threonine phosphatase known to dephosphorylate activated SMAD2/3 and terminate canonical TGF-β responses (Lin et al., 2006), it was selected for subsequent mechanistic validation.

We first examined whether MHE modulates the physical association between PPM1A and SMAD2/3. In HSC-LX2 cells expressing wild-type PPM1A, co-immunoprecipitation confirmed interactions between PPM1A and SMAD2/3 under basal conditions. TGF-β stimulation weakened these interactions, whereas MHE co-treatment restored the PPM1A-SMAD2/3 association toward basal levels (Figure 4B). Notably, MHE restored PPM1A-SMAD association without affecting total PPM1A protein abundance, suggesting modulation of complex assembly rather than altered protein expression. These findings support a model in which MHE counteracts TGF-β-associated disruption of the PPM1A-SMAD complex, consistent with enhanced signal termination.

To determine whether PPM1A catalytic activity contributes to MHE-mediated regulation of SMAD phosphorylation, we compared wild-type PPM1A with two catalytically inactive mutants (R174G and D239N) (Jackson et al., 2003). Wild-type PPM1A decreased SMAD2/3 phosphorylation, and this effect was further enhanced by MHE. In contrast, neither catalytic mutant reduced SMAD2/3 phosphorylation, and MHE did not attenuate SMAD phosphorylation in mutant-expressing cells (Figure 4C). Total SMAD2/3 abundance remained comparable across conditions, indicating preferential regulation of phosphorylation state rather than protein expression. These data indicate that PPM1A phosphatase activity is required for MHE-associated reduction of SMAD2/3 phosphorylation. Finally, we performed loss-of-function experiments to test whether endogenous PPM1A is required for the anti-fibrotic effects of MHE. Silencing of PPM1A abolished the ability of MHE to reduce SMAD2/3 phosphorylation, as confirmed by quantitative analysis (Figure 4D). Moreover, PPM1A knockdown eliminated MHE-mediated reductions in fibrotic markers (ACTA2, COL1A1, and FN) at both protein and mRNA levels (Figures 4E,F). Together, these findings identify PPM1A as a required regulatory node linking MHE treatment to restoration of SMAD2/3 dephosphorylation dynamics and suppression of TGF-β-driven HSC activation.

### 3.5 Isolation and structural elucidation of phytochemicals from MHE

**FIGURE 5:**
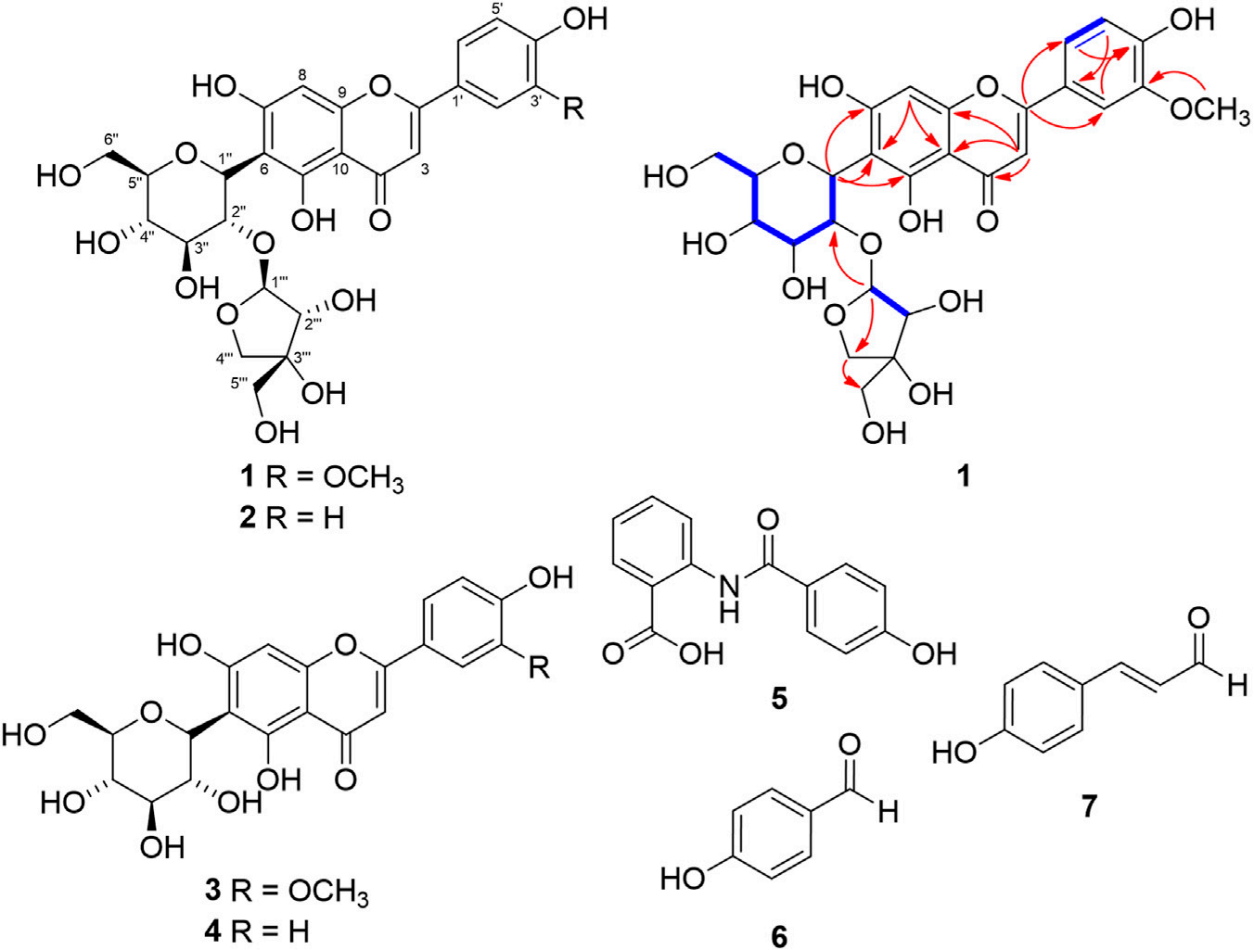
The chemical structures of all compounds (1–7) isolated from MHE and key COSY (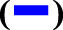) and HMBC (→) correlations of melandryoside.

After confirming the potent anti-fibrotic activity of MHE in *in vitro* and *in vivo* models, we sought to isolate the active phytochemicals responsible for its biological efficacy. This led to the isolation and structural elucidation of seven compounds (labeled 1–7) through a systematic combination of column chromatography and C_18_ reversed-phase HPLC (Figure 5). melandryoside was isolated as a yellow amorphous powder, and its positive-ion mode HR-ESIMS data exhibited an [M + H]^+^ ion peak at *m/z* 595.1654 (calc. for C_27_H_31_O_15_, 595.1657), which suggests that the molecular formula of melandryoside was C_27_H_30_O_15_. Based on the ^1^H NMR data (Supplementary Figure S1; Table 2), melandryoside possessed a C-6 substituted flavone skeleton of a chrysoeriol-type aglycone, as indicated by one methoxy group [*δ*_H_ 3.97 (3H, s, OCH_3_-3′)] and five aromatic protons [*δ*_H_ 6.52 (1H, s, H-8), 6.65 (1H, s, H-3), 6.94 (1H, d, *J* = 8.5 Hz, H-5′), 7.49 (1H, m, H-2′), and 7.51 (1H, d, *J* = 8.5 Hz, H-6′)] (Plazonić et al., 2009).

The existence of two sugar moieties in melandryoside was supported by two anomeric proton signals [*δ*_H_ 4.95 (1H, d, *J* = 10.0 Hz, H-1″) and 5.21 (1H, d, *J* = 1.5 Hz, H-1‴)], along with a series of oxygenated proton signals ranging from *δ*_H_ 3.27 to *δ*_H_ 3.94. These sugar units were further identified as glucose and apiose by analysis of ^1^H–^1^H COSY and HMBC spectra (Supplementary Figures S2, S5). The HSQC spectrum revealed correlations between the anomeric protons and their corresponding carbons [*δ*_c_ 72.1 (C-1″) and 108.4 (C-1‴)] (Supplementary Figure S4). In particular, the chemical shift of C-1″ provided definitive evidence for a *C*-glycosyl bond, distinguishing it from typical *O*-glycosides (Wang et al., 2011). Furthermore, a *C*-glucosyl moiety was inferred from the distinctive chemical shifts of the glucosyl unit *δ*_c_ 72.1 (C-1″), 81.2 (C-2″), 78.8 (C-3″), 72.2 (C-4″), 81.8 (C-5″), and 61.2 (C-6″). Based on the HMBC correlation from H-1‴ to C-2'' (*δ*_c_ 81.2), the apiose residue was positioned to the C-2″ position of the glucose unit. In the MS/MS spectrum, the fragment ion peak at *m/z* 463.12, corresponding to [(M + H) – 132]^+^, indicated the neutral loss of an apiosyl residue (Supplementary Figure S8). This fragmentation pattern confirms that melandryoside is a flavone diglycoside possessing a terminal pentosyl unit.

Detailed interpretation of the remaining 2D NMR correlations suggested that the chemical structure of melandryoside was similar to that of apigenin 6-*C*-[*β*-D-apiofuranosyl (1→2)]-*β*-D-glucopyranoside, differing only by the additional substitution of a methoxy group at C-3' (Tsolmon et al., 2021). The presence of a methoxy group at C-3′ of melandryoside was confirmed by HMBC correlation from 3′-OCH_3_ to C-3'. The D-configuration of this terminal apiose was validated by the positive specific rotation ([α]+7.5) of the sugar fraction obtained after acid hydrolysis (Jung et al., 2004). Consequently, melandryoside was identified as chrysoeriol 6-*C*-[*β*-D-apiofuranosyl-(1→2)]-*β*-D-glucopyranoside and was assigned the trivial name melandryoside (Figure 5).

The other compounds isolated from *M. firmum* were identified as apigenin-6-*C*-[*β*-D-apiofuranosyl-(1→2)]-*β*-D-glucopyranoside (2), chrysoeriol 6-C-glucoside (3), apigenin-6-C-glucoside (4), *N*-(4-hydroxybenzoyl) anthranilic acid (5), 4-hydroxybenzaldehyde (6), and *trans*-*p*-coumaraldehyde (7) upon comparison with spectroscopic data from previous literature and LC/MS analysis. Compound 5, originally known only as a synthetic derivative, was identified as a naturally occurring compound in our analysis.

### 3.6 Melandryoside recapitulates the anti-fibrotic effects of MHE through restoration of the PPM1A-SMAD regulatory complex

**FIGURE 6:**
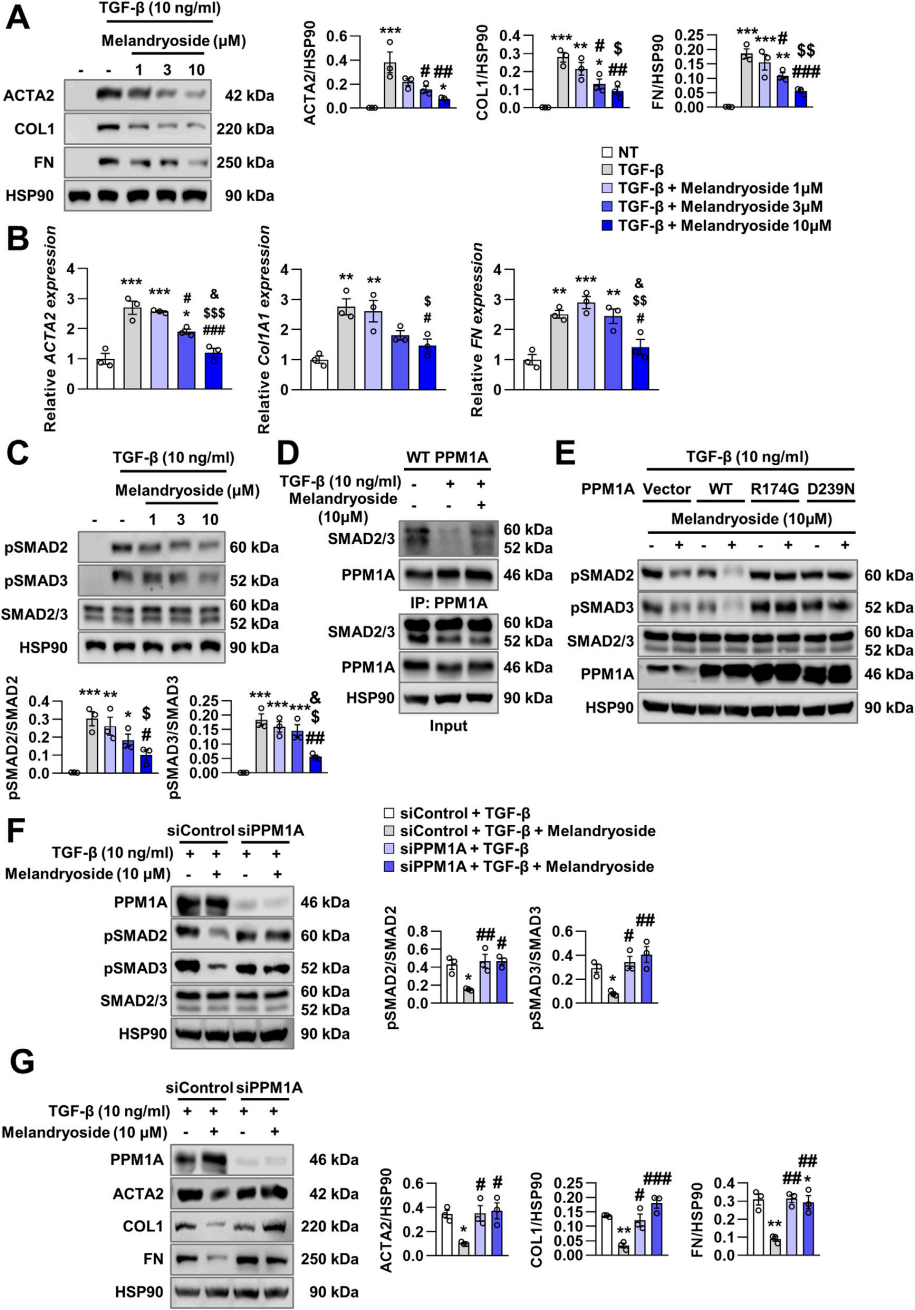
Regulation of fibrotic marker expression by the MHE-derived compound melandryoside in HSC-LX2 cells. **(A)** Immunoblot and quantitative analysis of ACTA2, COL1, and FN expression. **(B)** Relative mRNA expression of *ACTA2*, *COL1A1*, and *FN*. **(C)** Immunoblot and quantitative analysis of key proteins of the SMAD signaling pathway. **(D)** HSC-LX2 cells were transfected with wild-type (WT) PPM1A. The cells were then immunoprecipitated with anti-PPM1A antibody. Precipitates and cell lysates were subjected to immunoblotting for SMAD2/3 and PPM1A. **(E)** Immunoblot analysis of the expression of key proteins of the SMAD signaling pathway and PPM1A with WT PPM1A and two catalytically inactive PPM1A mutants (R174G and D239N). **(F,G)** HSC-LX2 cells were administered non-targeting siRNA (siControl) or PPM1A-targeting siRNA (siPPM1A). Immunoblot and quantitative analysis of the expression of **(F)** key proteins of the SMAD signaling pathway and **(G)** fibrotic markers and PPM1A. Data are presented as mean ± SEM (n = 3 per group). One-way ANOVA followed by Tukey multiple comparison test. *p < 0.05, **p < 0.01, ***p < 0.001 vs. NT; ^#^ *p <* 0.05, ^##^ *p <* 0.01, ^###^ *p <* 0.001 vs. TGF-β; ^$^ *p <* 0.05, ^$$^ *p <* 0.01, ^$$$^ *p <* 0.001 vs. TGF-β + Melandryoside 1µM; ^&^ *p <* 0.05 vs. TGF-β + Melandryoside 3 µM.

We next examined whether melandryoside reproduces the anti-fibrotic phenotype of MHE in HSC-LX2 cells and whether it converges on the same PPM1A-mediated SMAD regulatory axis identified in Figure 4. We first assessed the ability of melandryoside to modulate the fibrogenic activation program induced by sustained TGF-β exposure. Under prolonged TGF-β stimulation, melandryoside reduced canonical markers of HSC activation, including ACTA2 (α-SMA), COL1A1, and FN, in a dose-dependent manner at the protein level, as supported by quantitative analysis (Figure 6A). Concordantly, melandryoside decreased the corresponding fibrogenic transcripts (Figure 6B), indicating that the purified constituent is sufficient to suppress TGF-β-driven profibrotic responses.

Because these fibrotic outputs are regulated by canonical SMAD signaling, we next examined early pathway activation. Short pretreatment of HSC-LX2 cells with melandryoside followed by brief TGF-β challenge attenuated phosphorylation of both SMAD2 and SMAD3 in a concentration-dependent manner, as quantified by immunoblot analysis (Figure 6C). This finding links the reduction of fibrogenic gene expression by melandryoside with decreased proximal SMAD2/3 activation, consistent with the signaling phenotype observed with MHE (Figure 1D).

We next directly tested whether melandryoside engages the PPM1A-SMAD regulatory module identified for MHE. Co-immunoprecipitation experiments showed that TGF-β weakened the association between PPM1A and the SMAD complex, whereas melandryoside restored this interaction (Figure 6D), consistent with re-engagement of a SMAD termination complex. To determine whether PPM1A enzymatic function contributes to this signaling outcome, we assessed catalytically inactive PPM1A mutants. Unlike wild-type PPM1A, the catalytic mutants failed to support melandryoside-associated suppression of SMAD2/3 phosphorylation (Figure 6E), indicating that PPM1A phosphatase activity is required for this response. Silencing of PPM1A abolished melandryoside-induced reductions in SMAD2/3 phosphorylation (Figure 6F) and eliminated melandryoside-mediated suppression of fibrotic markers with corresponding quantitative analysis (Figure 6G). Together, these data demonstrate that melandryoside reproduces the principal anti-fibrotic signaling effects of MHE in suppressing TGF-β-driven HSC activation, and that these responses require a PPM1A-dependent SMAD2/3 dephosphorylation mechanism, thereby linking extract-level activity to a defined bioactive constituent.

### 3.7 Melandryoside attenuates palmitate-induced metabolic stress by reducing lipid accumulation and oxidative stress

**FIGURE 7:**
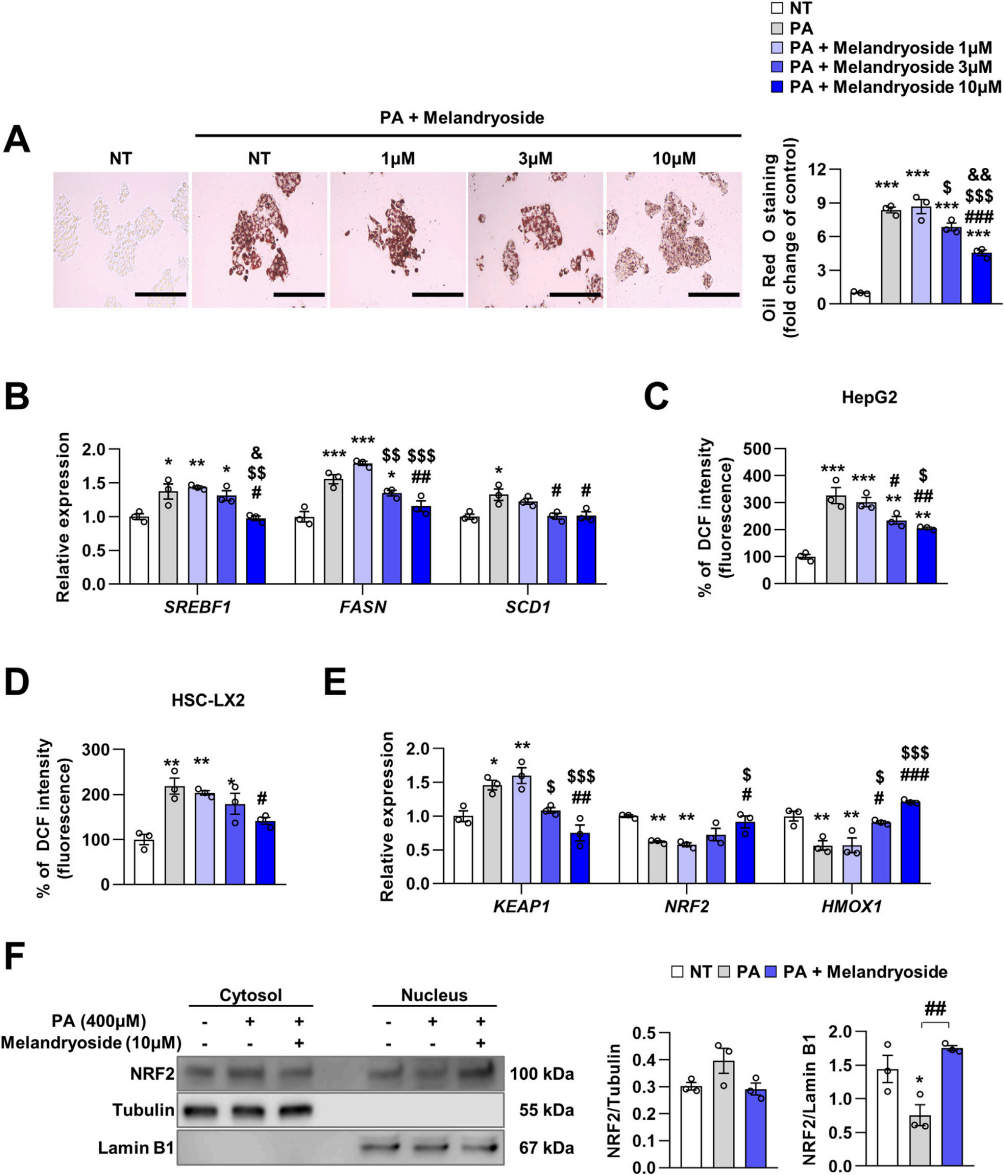
Modulation of palmitic acid-induced lipid accumulation and oxidative stress by melandryoside in hepatocytes. **(A)** Representative Oil Red O staining and quantification of intracellular lipid accumulation in HepG2 cells (scale bar, 100 μm). **(B)** Relative mRNA expression of *de novo* lipogenesis-related genes in HepG2 cells. **(C)** Intracellular ROS levels in HepG2 cells. **(D)** Intracellular ROS levels in HSC-LX2 cells. **(E)** Relative mRNA expression of oxidative stress-related genes in HepG2 cells. **(F)** Immunoblot and quantitative analysis of nuclear and cytoplasmic NRF2 expression in HepG2 cells. Data are presented as mean ± SEM (n = 3 per group). One-way ANOVA followed by Tukey multiple comparison test. *p < 0.05, **p < 0.01, ***p < 0.001 vs. NT; ^#^ *p <* 0.05, ^##^ *p <* 0.01, ^###^ *p <* 0.001 vs. PA; ^$^ *p <* 0.05, ^$$^ *p <* 0.01, ^$$$^ *p <* 0.001 vs. PA + Melandryoside 1µM; ^&^ *p <* 0.05, ^&&^ *p <* 0.01 vs. PA + Melandryoside 3 µM.

Having established that melandryoside reproduces the anti-fibrotic actions of MHE in HSCs (Figure 6), we next examined whether melandryoside also recapitulates the hepatoprotective arm of MHE under lipotoxic stress. To this end, we used a PA-induced model of lipid overload and oxidative injury and assessed intracellular lipid accumulation, lipogenic transcriptional programs, and oxidative stress readouts in hepatocytes, with parallel ROS assessment in stellate cells. PA exposure markedly increased intracellular lipid droplet accumulation in both HepG2 and FL83B cells. Co-treatment with melandryoside reduced Oil Red O-positive lipid deposition, and quantitative analysis demonstrated a dose-dependent decrease in lipid accumulation in both cell lines (Figure 7A; Supplementary Figure S9). To determine whether this reduction was accompanied by modulation of lipid anabolic programs rather than an isolated staining change, we measured transcripts involved in *de novo* lipogenesis. Melandryoside decreased the expression of lipogenesis-associated genes in PA-treated HepG2 cells (Figure 7B), consistent with attenuation of PA-driven lipogenic responses.

Because lipid overload can propagate fibrogenic signaling through oxidative stress, we next assessed intracellular ROS using a DCFH-DA assay. PA increased ROS levels relative to untreated controls, whereas melandryoside reduced PA-induced ROS generation in HepG2 cells (Figure 7C). Notably, a comparable reduction in ROS was observed in HSC-LX2 cells (Figure 7D), indicating that melandryoside modulates oxidative stress not only in hepatocytes but also in fibrogenic effector cells. To further evaluate broader oxidative stress-associated responses, we quantified ROS-related transcripts. Melandryoside decreased *KEAP1* expression while increasing *NRF2* and *HMOX1* transcripts compared with PA treatment alone (Figure 7E). To further assess NRF2 activation, nuclear and cytoplasmic fractions were analyzed by immunoblotting. PA modestly increased cytoplasmic NRF2 and significantly decreased nuclear NRF2 accumulation relative to NT cells. Melandryoside decreased cytoplasmic NRF2 and increased nuclear NRF2 accumulation relative to PA-treated cells, consistent with restoration of NRF2 nuclear translocation (Figure 7F). Collectively, these results indicate that melandryoside is sufficient to reproduce the principal protective metabolic effects of MHE by reducing PA-induced lipid accumulation, lipogenic gene activation, and ROS-associated stress signaling, while restoring NRF2-dependent antioxidant responses through recovery of NRF2 nuclear translocation in hepatocytes. These effects were accompanied by parallel attenuation of ROS in stellate cells, consistent with reduced upstream lipotoxic and oxidative cues that contribute to fibrogenic activation during MASH progression.

## 4 Discussion

MASH is increasingly recognized as a fibrosis-driven disease in which liver-related outcomes and mortality are more strongly associated with fibrosis stage than with steatosis or inflammatory activity alone (Zhu and Cai, 2025). The recent accelerated FDA approval of the THR-β agonist resmetirom for noncirrhotic MASH with F2-F3 fibrosis represents important clinical progress; however, histological responses remain incomplete in a substantial proportion of patients, and fibrosis regression is not universal (Zhu and Cai, 2025; Kokkorakis et al., 2024). Likewise, incretin-based therapies, including GLP-1-directed approaches, provide meaningful metabolic benefits and signals of steatohepatitis improvement, yet anti-fibrotic responses remain variable across studies and patient populations (Sanyal et al., 2025). These observations highlight the need for complementary therapeutic strategies that simultaneously reduce upstream metabolic injury and restrain downstream fibrogenic signaling.

The pathological processes examined in this study—lipid metabolism, oxidative stress, inflammation, and fibrosis—are mechanistically interconnected rather than independent. Hepatocellular lipotoxicity promotes oxidative stress and inflammatory signaling, creating a microenvironment that drives hepatic stellate cell activation and subsequent fibrogenesis. Within this pathogenic network, our findings suggest that MHE and melandryoside act at two coupled nodes: they attenuate upstream lipotoxic and oxidative injury, thereby reducing the profibrogenic cues delivered to hepatic stellate cells, while simultaneously reinforcing PPM1A-dependent termination of canonical SMAD2/3 signaling. This integrated mechanism provides a unifying framework linking the coordinated improvements observed across lipid metabolism, oxidative stress, inflammation, and fibrosis.

A major strength of the present study is the integration of hepatocyte, hepatic stellate cell, and *in vivo* data into a coherent pharmacological framework. The present study was primarily designed to define the molecular mechanism by which Melandrii Herba and its active constituent exert anti-fibrotic activity, with the *in vivo* experiment serving to confirm disease-relevant efficacy in a proof-of-concept manner. In hepatocytes, MHE and melandryoside reduced palmitate-induced lipid accumulation, suppressed *de novo* lipogenic programs, and attenuated oxidative stress, indicating protection against lipotoxic injury. Functionally, both MHE and melandryoside promoted NRF2 nuclear translocation, consistent with activation of the KEAP1/NRF2/HMOX1 antioxidant axis and with the reduction in ROS observed in hepatocytes and hepatic stellate cells. These effects are relevant because hepatocellular stress promotes inflammatory and pro-fibrogenic crosstalk within the liver microenvironment (Bansal and Bansal, 2024). In HSCs, MHE and melandryoside suppressed canonical TGF-β signaling, as reflected by reduced SMAD2/3 phosphorylation and lower expression of profibrotic markers (Meng et al., 2016; Zhu and Cai, 2025). Consistent with these cellular findings, oral administration of MHE improved steatosis, liver injury markers, inflammatory responses, and fibrosis in CDAHFD-fed mice. Collectively, these data support a multi-target mode of action aligned with the coupled metabolic and fibrotic features of MASH.

Our findings also extend prior pharmacological observations on Melandrii Herba into a MASH-relevant metabolic-fibrotic context. Previous work showed that *Melandryum firmum* extract alleviates obesity-associated metabolic abnormalities and hepatic lipid accumulation in high-fat diet-fed mice (Kim et al., 2020), whereas Melandrii Herba extract suppresses inflammatory signaling through inhibition of NF-κB/MAPK pathways and induction of HO-1 (Jeong et al., 2016). Additional studies demonstrated protection against oxidative stress-associated cellular injury and suggested a favorable preliminary safety profile in toxicological evaluation (Lee et al., 2017; Park et al., 2016). The present study links these previously separate observations—anti-obesity, anti-inflammatory, and cytoprotective effects—to a unified disease framework in which Melandrii Herba attenuates both upstream metabolic injury and downstream fibrogenic signaling in MASH.

Another important advance is the identification of melandryoside as a mechanistically relevant bioactive constituent of MHE. This finding is meaningful in the context of phytotherapeutic development because it provides an active-principle bridge between extract-level activity and molecular mechanism. Our data do not imply that melandryoside is the sole active constituent within MHE; rather, they establish that melandryoside is sufficient to reproduce the principal anti-lipotoxic and anti-fibrotic cellular effects of the extract. This positions melandryoside as both a mechanistic lead constituent and a plausible marker compound for future quality control of MHE. This interpretation is also consistent with prior phytochemical studies showing that Melandrii Herba contains multiple bioactive constituents, including sterol- and triterpenoid-related molecules such as α-spinasterol and ursolic acid, which possess anti-inflammatory and hepatometabolic activities (Zheng et al., 2008; Majeed et al., 2022; Lee et al., 2022; Kwon et al., 2018).

A central mechanistic insight of this study is that the anti-fibrotic actions of MHE and melandryoside depend on reinforcement of SMAD signal termination centered on PPM1A, a serine/threonine phosphatase that dephosphorylates SMAD2/3 and limits canonical TGF-β signaling (Lin et al., 2006; Wang et al., 2010). Through an interactome-guided prioritization strategy, we intersected SMAD2-and SMAD3-associated protein networks and identified PPM1A as the only phosphatase shared by both interaction sets, supporting its plausibility as a common negative regulator of SMAD signaling. In HSCs, TGF-β weakened the association between PPM1A and the SMAD complex, whereas MHE and melandryoside restored this interaction and reduced SMAD2/3 phosphorylation in a manner consistent with accelerated signal resolution (Lin et al., 2006). Genetic silencing of PPM1A abolished the anti-fibrotic effects of MHE and melandryoside, and catalytically inactive PPM1A mutants failed to support suppression of SMAD2/3 phosphorylation, indicating that PPM1A catalytic activity is required for these responses (Lin et al., 2006; Jackson et al., 2003; Cheng et al., 2024). Notably, MHE restored the PPM1A-SMAD association without altering total PPM1A abundance, suggesting modulation of complex assembly rather than transcriptional upregulation (Lin et al., 2006; Yang et al., 2022).

This concept is supported by previous studies showing that SMAD dephosphorylation can be enhanced through regulation of phosphatase-SMAD interactions rather than by direct inhibition of upstream receptors (Deng et al., 2024). For example, the lncRNA GAS5 has been reported to promote PPM1A-SMAD3 association and accelerate SMAD3 dephosphorylation, demonstrating that pathway control can be achieved through regulation of complex formation without changing phosphatase abundance (Tang et al., 2020). In this context, our findings suggest that melandryoside may facilitate restoration of the PPM1A-SMAD complex under TGF-β stimulation, thereby promoting physiological signal termination. Nevertheless, direct ligand-target engagement and structural studies will be required to define the proximal molecular basis of this effect.

Conceptually, this mechanism differs from broad TGF-β pathway blockade, which has historically faced safety and tolerability concerns because of the pleiotropic roles of TGF-β in tissue repair and homeostasis (Deng et al., 2024). By reinforcing an endogenous negative-feedback checkpoint, MHE and melandryoside may provide a more selective anti-fibrotic approach capable of restraining pathogenic signaling while preserving broader pathway function. The identification of melandryoside as a bioactive constituent also provides a practical framework for phytotherapeutic development. This may support extract standardization using defined marker compounds and mechanism-linked pharmacodynamic monitoring, including SMAD2/3 phosphorylation signatures and fibrosis-associated readouts. Consistent with this view, re-engagement of the PPM1A-SMAD termination complex aligns with emerging pharmacological paradigms in which small molecules modulate protein-protein interactions to restore productive regulatory assemblies (Scott et al., 2016; Nada et al., 2024). This positioning may be particularly relevant in fibrotic disease, where sustained pathway resolution, rather than transient receptor-level inhibition, could provide a more durable means of reprogramming pathogenic signaling states.

Several limitations warrant consideration. CDAHFD robustly recapitulates steatohepatitis and fibrosis but incompletely models obesity-associated insulin resistance; therefore, validation in complementary metabolic models is warranted. Furthermore, the proximal mechanism by which MHE and melandryoside restore the PPM1A-SMAD complex remains unresolved and will require direct target-engagement and complex-assembly studies. In addition, although lipid droplet loss is a characteristic feature of hepatic stellate cell activation (Xu et al., 2005; Tsuchida and Friedman, 2017), intracellular lipid droplets were not reliably detectable in HSC-LX2 cells under the experimental conditions used in this study. Consequently, the present study could not determine whether MHE modulates lipid droplet dynamics during HSC activation, and this question should be addressed in future studies using primary quiescent hepatic stellate cells. Treatment was initiated after disease establishment to model a therapeutic setting; however, the present design did not include a histological baseline cohort at the time of treatment onset (week 8). Therefore, our findings should be interpreted as attenuation of disease progression under continued dietary challenge rather than reversal of pre-existing fibrosis, and formal demonstration of fibrosis regression will require baseline-controlled designs incorporating a treatment-onset cohort. Although pharmacological benchmark comparators were not included in the *in vivo* experiments, we performed additional *in vitro* comparisons using metformin as a mechanistic reference compound. Under TGF-β-stimulated conditions, MHE exhibited inhibitory effects on SMAD2/3 phosphorylation and fibrotic marker expression comparable to those observed with metformin (Supplementary Figures S10A, S10B), providing additional mechanistic support for its anti-fibrotic activity. Nevertheless, direct comparison with approved therapies for MASH, such as resmetirom, will require appropriately designed dose-ranging and exposure-matched *in vivo* studies to enable rigorous evaluation of relative therapeutic efficacy. Future studies incorporating such head-to-head comparisons will help establish the therapeutic potential of MHE and melandryoside relative to approved therapies for MASH. Further translational evaluation will also require characterization of exposure-response pharmacokinetics, tissue distribution, safety profiles, and confirmation in human-relevant fibrotic systems such as primary human HSCs, organoids, or precision-cut liver slices.

In summary, melandryoside, a bioactive constituent of Melandrii Herba, reproduced the principal metabolic and anti-fibrotic actions of the extract and suppressed fibrogenic signaling through reinforcement of PPM1A-dependent SMAD termination. These findings provide pharmacological support for the traditional medicinal use of Melandrii Herba, identify melandryoside as an active constituent, and highlight restoration of physiological signal resolution as a potential anti-fibrotic approach for MASH.

## 5 Conclusion

**FIGURE 8:**
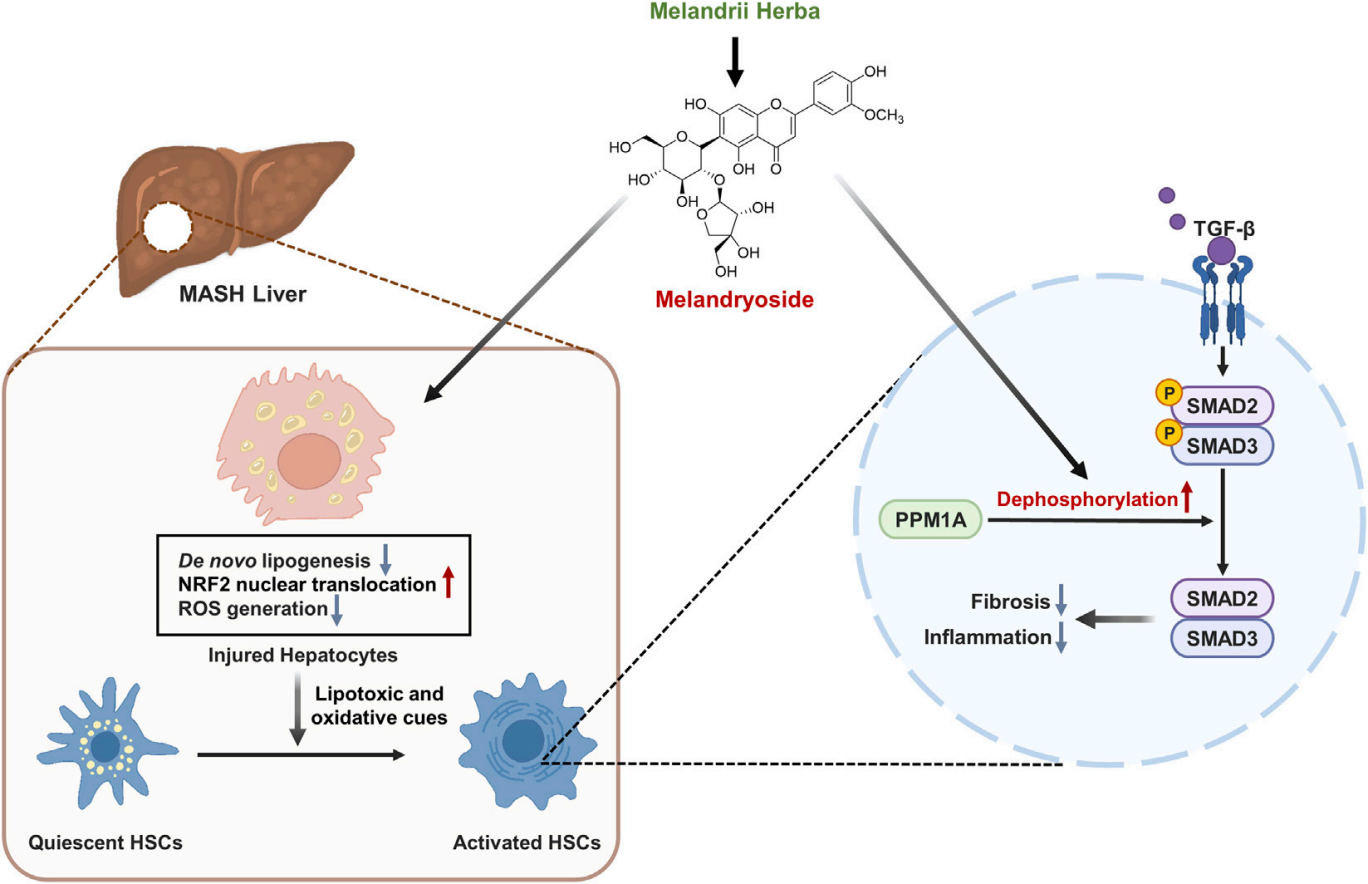
Schematic diagram of the mechanism of MHE and melandryoside attenuates MASH progression. MHE suppresses hepatocellular lipogenic and oxidative stress, thereby disrupting hepatocyte-driven activation of hepatic stellate cells. Melandryoside restores PPM1A-dependent SMAD2/3 termination to block TGF-β fibrogenic signaling. Together, these multi-node actions attenuate hepatic inflammation and fibrosis in MASH.

MHE attenuated hepatocellular lipotoxicity and oxidative stress while suppressing TGF-β-driven hepatic stellate cell activation. In CDAHFD-fed mice, MHE administered therapeutically after disease establishment attenuated steatosis, inflammatory injury, and fibrosis across key pathological features of MASH. Mechanistically, these protective effects were recapitulated by the purified constituent melandryoside and required PPM1A-mediated SMAD2/3 signal termination, thereby linking extract-level activity to a defined bioactive constituent and a mechanistically relevant anti-fibrotic checkpoint. Collectively, these findings provide pharmacological support for the traditional medicinal use of Melandrii Herba, establish melandryoside as an active constituent, and identify restoration of PPM1A-SMAD signaling control as a potential therapeutic approach for MASH (Figure 8).

## Data Availability

The original contributions presented in the study are included in the article/Supplementary Material, further inquiries can be directed to the corresponding authors.
