# Supplementary material for: Melandryoside attenuates steatohepatitis and fibrosis by restoring PPM1A-mediated SMAD signaling termination
Source: Front Pharmacol. 2026 Aug 5;17:1880176. doi: 10.3389/fphar.2026.1880176 (PMC13487451; doi:10.3389/fphar.2026.1880176)
Supplement: Supplementary file 1 [file Supplementaryfile1.docx]

Supplementary Material

**Melandryoside attenuates steatohepatitis and fibrosis by restoring PPM1A-mediated SMAD signaling termination**

Yo Han Lee^1,#^, Youngsang Nam^2,#^, Kieun Park^1^, Haneul Ji^1^, Ji Won Kim^1^,Sooyeon Cho^1^, Na Gyeong Kim^1^, Kwangho Kim^1^, Jiwon Yoo^1^, Ji Young Lee^1^, Chang-Hwan Bae^3^, Eunsoo Kim^2^, Eun Seo Jang^2^, Won Kon Kim^4^, Hwayoung Yun^2^, Seoung Rak Lee^2,^**^*^**, Jun Hong Park^5,^**^*^**, Jang Hyun Choi^1,6,^**^*^**

^1^Department of Biological Sciences, Ulsan National Institute of Science and Technology (UNIST), Ulsan 44919, Republic of Korea

^2^College of Pharmacy and Research Institute for Drug Development, Pusan National University, Busan 46241, Republic of Korea

^3^Herbal Medicine Resources Research Center, Korea Institute of Oriental Medicine, Naju, Jeollanam-do, 58245, Republic of Korea

^4^Metabolic Regulation Research Center, Korea Research Institute of Bioscience and Biotechnology (KRIBB), Daejeon 34141, Republic of Korea

^5^College of Veterinary Medicine, Jeonbuk National University, Iksan-si, Jeollabuk-do 54596, Republic of Korea

^6^Metadium Therapeutics, Ulsan 44919, Republic of Korea

#These authors contributed equally to this work.

# 1 Supplementary Figures and Tables

**Figure S1.** The HR-ESIMS and HPLC data (254 nm) of melandryoside

**Figure S2.** The ^1^H NMR spectrum of melandryoside (CD_3_OD, 400 MHz)

**Figure S3.** The ^1^H-^1^H COSY spectrum of melandryoside (CD_3_OD)

**Figure S4.** The HSQC spectrum of melandryoside (CD_3_OD)

**Figure S5.** The HMBC spectrum of melandryoside (CD_3_OD)

**Figure S6.** MHE reduces PA-induced lipid accumulation in FL83B cells

**Figure S7.** Schematic overview of SMAD2/3-binding proteins

**Figure S8.** The MS/MS spectrum in positive of melandryoside

**Figure S9.** Melandryoside decreases lipid accumulation in PA-treated FL83B cells

**Figure S10.** Comparison of the anti-fibrotic effects of MHE, melandryoside, and metformin in TGF-β-stimulated HSC-LX2 cells

**Figure S1.** The HR-ESIMS and HPLC data (254 nm) of melandryoside

HPLC analysis was performed under the following conditions: an Agilent 1260 HPLC system equipped with a 3.5 μm C_18_ column (3.0 mm × 150 mm) was used. The mobile phase consisted of water (eluent A) and MeOH containing 0.1% formic acid (eluent B). The flow rate was maintained at 0.3 mL/min, and the proportion of eluent B was increased linearly from 10% to 100% over 15 min. The total run time was 20 min, with the final 5 min used as a column-washing step at 100% eluent B.
**Figure S2.** The ^1^H NMR spectrum of melandryoside (CD_3_OD, 400 MHz)

**Figure S3.** The ^1^H-^1^H COSY spectrum of melandryoside (CD_3_OD)

**Figure S4.** The HSQC spectrum of melandryoside (CD_3_OD)

**Figure S5.** The HMBC spectrum of melandryoside (CD_3_OD)

**Figure S6.** MHE reduces PA-induced lipid accumulation in FL83B cells

Representative Oil Red O staining and quantification of intracellular lipid accumulation in FL83B cells (scale bar, 100 μm). Data are presented as mean ± SEM (n = 3 per group). One-way ANOVA followed by Tukey multiple comparison test. ***p <* 0.01, ****p <* 0.001 vs NT; ^##^*p <* 0.01, ^###^*p <* 0.001 vs PA; ^$^*p <* 0.05, ^$$$^*p <* 0.001 vs PA + MHE 25µg/ml; ^&^*p <* 0.05 vs PA + MHE 50µg/ml.

**Figure S7.** Schematic overview of SMAD2/3-binding proteins

Functional classification of shared SMAD2/3-interacting proteins identifies PPM1A as the only phosphatase.

**Figure S8.** The MS/MS spectrum in positive of melandryoside

**Figure S9.** Melandryoside decreases lipid accumulation in PA-treated FL83B cells

Representative Oil Red O staining and quantification of intracellular lipid accumulation in FL83B cells (scale bar, 100 μm). Data are presented as mean ± SEM (n = 3 per group). One-way ANOVA followed by Tukey multiple comparison test. **p <* 0.05, ****p <* 0.001 vs NT; ^#^*p <* 0.05, ^###^*p <* 0.001 vs PA; ^$^*p <* 0.05, ^$$$^*p <* 0.001 vs PA + Melandryoside 1µM; ^&^*p <* 0.05 vs PA + Melandryoside 3µM.

**Figure S10.** Comparison of the anti-fibrotic effects of MHE, melandryoside, and metformin in TGF-β-stimulated HSC-LX2 cells

(A) Immunoblot analysis of key proteins of the SMAD signaling pathway in TGF-β-stimulated HSC-LX2 cells treated with MHE (100µg/ml), melandryoside (10µM), or metformin (1mM).

(B) Immunoblot analysis of ACTA2, COL1, and FN expression in TGF-β-stimulated HSC-LX2 cells treated with MHE (100µg/ml), melandryoside (10µM), or metformin (1mM).
